# Supplementary material for: Targeted and tailored pharmacist-led intervention to improve adherence to antihypertensive drugs among patients with type 2 diabetes in Indonesia: study protocol of a cluster randomised controlled trial
Source: BMJ Open. 2020 Jan 6;10(1):e034507. doi: 10.1136/bmjopen-2019-034507 (PMC6955569; doi:10.1136/bmjopen-2019-034507)
Supplement: Supplementary data [file bmjopen-2019-034507supp001.pdf]

APPENDIX

A. SPIRIT checklist

Section/item	Item No	Description	Reported on page
**Administrative information**			
Title	1	Descriptive title identifying the study design, population, interventions, and, if applicable, trial acronym	1
Trial registration	2a	Trial identifier and registry name. If not yet registered, name of intended registry	2
	2b	All items from the World Health Organization Trial Registration Data Set	4-20
Protocol version	3	Date and version identifier	NA
Funding	4	Sources and types of financial, material, and other support	17
Roles and responsibilities	5a	Names, affiliations, and roles of protocol contributors	1, 17
	5b	Name and contact information for the trial sponsor	NA
	5c	Role of study sponsor and funders, if any, in study design; collection, management, analysis, and interpretation of data; writing of the report; and the decision to submit the report for publication, including whether they will have ultimate authority over any of these activities	17
	5d	Composition, roles, and responsibilities of the coordinating centre, steering committee, endpoint adjudication committee, data management team, and other individuals or groups overseeing the trial, if applicable (see Item 21a for data monitoring committee)	NA

Alfian SD, *et al. BMJ Open* 2020; 10:e034507. doi: 10.1136/bmjopen-2019-034507

**Introduction**			
Background and rationale	6a	Description of research question and justification for undertaking the trial, including summary of relevant studies (published and unpublished) examining benefits and harms for each intervention	4-5
	6b	Explanation for choice of comparators	10
Objectives	7	Specific objectives or hypotheses	5
Trial design	8	Description of trial design including type of trial (eg, parallel group, crossover, factorial, single group), allocation ratio, and framework (eg, superiority, equivalence, non-inferiority, exploratory)	6,7
**Methods: Participants, interventions, and outcomes**			
Study setting	9	Description of study settings (eg, community clinic, academic hospital) and list of countries where data will be collected. Reference to where list of study sites can be obtained	6
Eligibility criteria	10	Inclusion and exclusion criteria for participants. If applicable, eligibility criteria for study centres and individuals who will perform the interventions (eg, surgeons, psychotherapists)	6,7
Interventions	11a	Interventions for each group with sufficient detail to allow replication, including how and when they will be administered	7-10
	11b	Criteria for discontinuing or modifying allocated interventions for a given trial participant (eg, drug dose change in response to harms, participant request, or improving/worsening disease)	NA
	11c	Strategies to improve adherence to intervention protocols, and any procedures for monitoring adherence (eg, drug tablet return, laboratory tests)	14
	11d	Relevant concomitant care and interventions that are permitted or prohibited during the trial	7
Outcomes	12	Primary, secondary, and other outcomes, including the specific measurement variable (eg, systolic blood pressure), analysis metric (eg, change from baseline, final value, time to event), method of aggregation (eg, median, proportion), and time point for each outcome. Explanation of the clinical relevance of chosen efficacy and harm outcomes is strongly recommended	11-13
Participant timeline	13	Time schedule of enrolment, interventions (including any run-ins and washouts), assessments, and visits for participants. A schematic diagram is highly recommended (see Figure)	Figure 1

Alfian SD, *et al. BMJ Open* 2020; 10:e034507. doi: 10.1136/bmjopen-2019-034507

Sample size	14	Estimated number of participants needed to achieve study objectives and how it was determined, including clinical and statistical assumptions supporting any sample size calculations	14
Recruitment	15	Strategies for achieving adequate participant enrolment to reach target sample size	14
**Methods: Assignment of interventions (for controlled trials)**			
Allocation:			
Sequence generation	16a	Method of generating the allocation sequence (eg, computer-generated random numbers), and list of any factors for stratification. To reduce predictability of a random sequence, details of any planned restriction (eg, blocking) should be provided in a separate document that is unavailable to those who enrol participants or assign interventions	7
Allocation concealment mechanism	16b	Mechanism of implementing the allocation sequence (eg, central telephone; sequentially numbered, opaque, sealed envelopes), describing any steps to conceal the sequence until interventions are assigned	7
Implementation	16c	Who will generate the allocation sequence, who will enrol participants, and who will assign participants to interventions	7
Blinding (masking)	17a	Who will be blinded after assignment to interventions (eg, trial participants, care providers, outcome assessors, data analysts), and how	7
	17b	If blinded, circumstances under which unblinding is permissible, and procedure for revealing a participant's allocated intervention during the trial	NA
**Methods: Data collection, management, and analysis**			
Data collection methods	18a	Plans for assessment and collection of outcome, baseline, and other trial data, including any related processes to promote data quality (eg, duplicate measurements, training of assessors) and a description of study instruments (eg, questionnaires, laboratory tests) along with their reliability and validity, if known. Reference to where data collection forms can be found, if not in the protocol	11-14
	18b	Plans to promote participant retention and complete follow-up, including list of any outcome data to be collected for participants who discontinue or deviate from intervention protocols	11, 14
Data management	19	Plans for data entry, coding, security, and storage, including any related processes to promote data quality (eg, double data entry; range checks for data values). Reference to where details of data management procedures can be found, if not in the protocol	15

Alfian SD, *et al. BMJ Open* 2020; 10:e034507. doi: 10.1136/bmjopen-2019-034507

Statistical methods	20a	Statistical methods for analysing primary and secondary outcomes. Reference to where other details of the statistical analysis plan can be found, if not in the protocol	15
	20b	Methods for any additional analyses (eg, subgroup and adjusted analyses)	15
	20c	Definition of analysis population relating to protocol non-adherence (eg, as randomised analysis), and any statistical methods to handle missing data (eg, multiple imputation)	15
**Methods: Monitoring**			
Data monitoring	21a	Composition of data monitoring committee (DMC); summary of its role and reporting structure; statement of whether it is independent from the sponsor and competing interests; and reference to where further details about its charter can be found, if not in the protocol. Alternatively, an explanation of why a DMC is not needed	NA
	21b	Description of any interim analyses and stopping guidelines, including who will have access to these interim results and make the final decision to terminate the trial	NA
Harms	22	Plans for collecting, assessing, reporting, and managing solicited and spontaneously reported adverse events and other unintended effects of trial interventions or trial conduct	15
Auditing	23	Frequency and procedures for auditing trial conduct, if any, and whether the process will be independent from investigators and the sponsor	NA
**Ethics and dissemination**			
Research ethics approval	24	Plans for seeking research ethics committee/institutional review board (REC/IRB) approval	17
Protocol amendments	25	Plans for communicating important protocol modifications (eg, changes to eligibility criteria, outcomes, analyses) to relevant parties (eg, investigators, REC/IRBs, trial participants, trial registries, journals, regulators)	NA
Consent or assent	26a	Who will obtain informed consent or assent from potential trial participants or authorised surrogates, and how (see Item 32)	6
	26b	Additional consent provisions for collection and use of participant data and biological specimens in ancillary studies, if applicable	NA
Confidentiality	27	How personal information about potential and enrolled participants will be collected, shared, and maintained in order to protect confidentiality before, during, and after the trial	17

Alfian SD, *et al. BMJ Open* 2020; 10:e034507. doi: 10.1136/bmjopen-2019-034507

Declaration of interests	28	Financial and other competing interests for principal investigators for the overall trial and each study site	17
Access to data	29	Statement of who will have access to the final trial dataset, and disclosure of contractual agreements that limit such access for investigators	17
Ancillary and post-trial care	30	Provisions, if any, for ancillary and post-trial care, and for compensation to those who suffer harm from trial participation	NA
Dissemination policy	31a	Plans for investigators and sponsor to communicate trial results to participants, healthcare professionals, the public, and other relevant groups (eg, via publication, reporting in results databases, or other data sharing arrangements), including any publication restrictions	17
	31b	Authorship eligibility guidelines and any intended use of professional writers	NA
	31c	Plans, if any, for granting public access to the full protocol, participant-level dataset, and statistical code	NA
**Appendices**			
Informed consent materials	32	Model consent form and other related documentation given to participants and authorised surrogates	Appendix B, page 6
Biological specimens	33	Plans for collection, laboratory evaluation, and storage of biological specimens for genetic or molecular analysis in the current trial and for future use in ancillary studies, if applicable	NA

Alfian SD, *et al. BMJ Open* 2020; 10:e034507. doi: 10.1136/bmjopen-2019-034507

B. Participant information sheet and participant consent form

PARTICIPANT INFORMATION SHEET

"A tailored and targeted pharmacist intervention to improve adherence to antihypertensive drugs among patients with diabetes in Indonesia: a cluster randomized controlled trial"

This study is conducted by Sofa D. Alfian (PhD student at University of Groningen, the Netherlands), under daily supervision of Rizky Abdulah, PhD (Universitas Padjadjaran, Indonesia) and the supervision of Job F.M. van Boven, PhD (University Medical Center Groningen, Groningen, the Netherlands), Prof. Petra Denig (University Medical Center Groningen, Groningen, the Netherlands) and Prof. Eelko Hak (University of Groningen, Groningen, the Netherlands). You are invited to take part in a study that assesses different types of pharmacist services regarding the use of your antihypertensive drugs.

Objectives of the study

We want to compare the different types of pharmacist services on how you use your antihypertensive drugs, your blood pressure level, and your views about your antihypertensive drugs. We also want to know your opinion about the pharmacy service you received.

Participants

We are interested to include participants who:

- Are aged over 18 years
- Have type 2 diabetes
- Use antihypertensive drugs for at least three months

Procedure

The *Puskesmas* will be randomly assigned to different types of pharmacist services. The *Puskesmas* will have an equal chance of being placed in either group (like the flip of a coin). After you sign a written informed consent, you will receive guideline-based pharmacist counseling or patient-centred based counseling depending on your *Puskesmas*. Per *Puskesmas,* everyone will receive the same program. Your study participation will require three visits during the follow-up of 3 months with approximately up to 15-20 minutes per visit. We will

also ask you to complete a questionnaire. In addition, we will collect information from your medical record about your disease and other medication you are using.

Possible risks and inconvenience

There are no expected risks from participating in this study. However, up to 15-20 minutes of your time will be used during your regular visit to *Puskesmas*.

Benefits and compensation

There are no direct benefits of participation guaranteed to you in either group. Participation in either group will be at no cost to you. A modest compensation will be provided for your participation.

Participation and withdrawal from the study

Although it is important, your participation in this study is voluntary. You can change your mind at any time and you do not have to say why.

Confidentiality

All of the information you provide will be treated confidentially. A unique identifier will be used to match your data across your visits. Data will be stored securely and anonymously and only available to authorized individuals for analysis and reporting purpose only. Data will be published in a form that does not identify you in any way. Data will be stored for 15 years.

Whom to contact

If you have any concerns about this study that you think I can help you with, please feel free to contact me on: Sofa D. Alfian, phone: +62 85223 083 624, *email*: sofa.alfian@unpad.ac.id

If you would like to talk to someone who is not connected with the research, you may contact the Research Ethics Officer by *email:* etik.unpad@gmail.com

PARTICIPANT CONSENT FORM

I _______________________ (participant's name) agree to participate in the study "A tailored and targeted pharmacist intervention to improve adherence to antihypertensive drugs among patients with type 2 diabetes in Indonesia: a cluster randomized controlled trial" being conducted by Sofa D. Alfian of the University of Groningen for her PhD degree at the Groningen Research Institute of Pharmacy, Unit Pharmaco-Therapy, -Epidemiology & -Economics, The Netherlands.

By giving my consent, I acknowledge that:

- I have understood the Participant Information Sheet and have been given the opportunity to discuss the information and my involvement in the study.
- The procedures required for the study, the time involved (around 15-20 minutes per visit), and any inconvenience have been explained to me.
- I agree that information about my disease and other drugs I am using is collected from medical records, and is used in this study in a way that does not identify me.
- I understand that being in this study is voluntary.
- I understand that I am free to withdraw my participation from this study at any time I wish, without consequences, and without giving a reason.
- I agree that the research data gathered from this study may be published in a form that does not identify me in any way.
- I am aware that I can contact researcher (Sofa D. Alfian) if I have concerns about the research

Date ___/___/___

Signature,

Alfian SD, *et al. BMJ Open* 2020; 10:e034507. doi: 10.1136/bmjopen-2019-034507

C. Personalized leaflet

Name of patient : **Date:**
Agreed goal :
Date for next visit :

**Your blood pressure lowering drugs**			
Medicine name	When and how to take it?	What does it do?	Specific remarks

Reminders, habit-based strategies and/or involvement of family member

☐ Use pill boxes or a reminder app in your mobile phone.
☐ Make taking your blood pressure lowering drug(s) a part of your routine. Take it every day at around the same time. Choose a time that is easy to remember.

**Coping plan (first visit) ***	
IF it is (time of day):<br>……………………………………………….<br>And I am (where):<br>……………………………………………….<br>And I am (doing what):<br>……………………………………………….<br>THEN I will take (names of medication)<br>……………………………………………….<br>……………………………………………….	IF it is (time of day):<br>……………………………………………….<br>And I am (where):<br>……………………………………………….<br>And I am (doing what):<br>……………………………………………….<br>THEN I will take (names of medication)<br>……………………………………………….<br>……………………………………………….
**Follow-up after 1 month**	Succeeded ☐<br>If not, please explain why:<br>……………………………………………….

* Example: IF it is: first thing in the morning, and I am: in the kitchen, and I: am making my cup of tea, THEN: I will take my drug A

☐ Ask a family member to remind you to take your blood pressure lowering drug(s). I am going to ask: ………………………………….
☐ If you forgot to take the drug and it is less than 8 hours before your next dose, do not take a double dose of your blood pressure lowering drug(s) to make up for a forgotten dose. If it is more than 8 hours before your next dose, take the forgotten dose and take the next one as scheduled.

Alfian SD, *et al. BMJ Open* 2020; 10:e034507. doi: 10.1136/bmjopen-2019-034507

Knowledge about your blood pressure lowering drug(s)

- [ ] The purpose of your blood pressure lowering drug(s) is to reduce blood pressure level (short term effect) and to reduce the risk of developing heart disease in the future (long-term effect).
- [ ] Swallow your blood pressure lowering drug(s) with a drink of water.
- [ ] Common possible side effects of blood pressure lowering drug(s) are persistent dry cough, headache, dizziness, etc. If you get any side effects, please talk to your doctor or pharmacist. This includes any possible side effects not listed in this leaflet.

Motivation to take your blood pressure lowering drug(s)

- [ ] Your blood pressure lowering drug(s) only works when taken regularly as prescribed.
- [ ] Your blood pressure lowering drug(s) will not necessarily make you feel any different, but this does not mean it is not working.
- [ ] Do not stop taking your blood pressure lowering drug(s) without your doctor's consent unless you have an allergic reaction, e.g. rash or difficulty breathing. Abruptly stopping your blood pressure lowering drug(s) can cause a sudden increase in your blood pressure, which can be dangerous in some patients.
- [ ] Like all medicines, your blood pressure lowering drug(s) can cause side effects, but not everybody gets them. If you get any side effects, please talk to your doctor or pharmacist.
- [ ] Other:

____

____

____

Other drug-related problems

- [ ] If you have problem to refill your blood pressure lowering drug(s), please ask for help from your family or friend to accompany you to refill your medication.
- [ ] Other:

____

____

Alfian SD, *et al. BMJ Open* 2020; 10:e034507. doi: 10.1136/bmjopen-2019-034507

D. <u>Counseling protocol in the intervention group</u>

Baseline session

1. **Opening**

   *I would like to start with reviewing some of your medications and then we can talk about your experiences of taking them. (*Complete the "Your blood pressure lowering drugs' list at the top of the Personalized Leaflet*)*

2. **Identify any possible barriers using the <u>flowchart at baseline</u> and provide the intervention based on the <u>adherence intervention wheel</u>**

   Try to find out whether there are any barriers for not using the blood pressure lowering drug(s). Ask more if something is not clear. Once any barriers have been identified, deliver the intervention accordingly.

   1. **Forgetfulness: setting coping plans and reminder tools**

      Do not try to come directly with solutions and answers. Let the patient formulate his/her plans.

      1. *How often do you forget to take your blood pressure lowering drug(s)?*
      2. *Do you know when or why it happens that you forget to take your blood pressure lowering drug(s)?*

         (Possible answers: being busy, not able to make taking the drug a part of their routine, difficult to remember when to take the drug, forgot to take the drug when traveling, or lack of family support. Patients may have other answers regarding the reason for forgetfulness, please provide and adapt the solution accordingly.)
      3. *What time do you usually take each of your blood pressure lowering drug(s)?*
      4. *What would you like to see different about your current situation?*

Alfian SD, *et al. BMJ Open* 2020; 10:e034507. doi: 10.1136/bmjopen-2019-034507

5. *I would start with a simple way for reminding you to take your medicines such as set an alarm on your mobile phone or set a coping plan.* (Give the personalized leaflet and ask patients to write down their plans in the box Reminders).
6. *Could you ask your family to remind you to take your blood pressure lowering drug(s) regularly (if so, ask patient to write down name of family member in the box Involvement of family member)?*
7. Explain what to do when a dose is forgotten, for example*: If you forget to take a dose of your blood pressure lowering drug, take it as soon as you remember. However, if it less than 8 hours before your next dose is scheduled, skip the missed dose(s) and go back to your regular dosing schedule. Do not take a double dose of your blood pressure lowering drug to make up for a forgotten dose.*

2. Lack of knowledge: counseling and education

1. *Do you understand why you have to take your blood pressure lowering drug(s)?*
   a. If yes: *Can you explain this to me?*
   b. If no or insufficient: explain the purpose of blood pressure lowering drug(s) regarding short (blood pressure) and long-term effects (prevention of heart attack and stroke); when relevant also explain which side effects can occur and how to deal with them when they occur. For example:

Alfian SD, *et al. BMJ Open* 2020; 10:e034507. doi: 10.1136/bmjopen-2019-034507

i. Short term effect: *The purpose of your blood pressure lowering drug(s) is to reduce your blood pressure by relaxing or widening your blood vessels, making your heartbeat more slowly, or flushing excess water and salt from the body through your pee.*

ii. Long term effect*: The purpose of your blood pressure lowering drug(s) is to reduce the risk of developing heart disease in the future, such as heart attack and stroke.*

2. *Do you understand how you have to take your blood pressure lowering drug(s)?*
   a. If yes: *Can you explain this to me?*
   b. If no or insufficient: Explain when to take and how much to take.
3. *Could you explain what we have talked about so far?*
4. *Do you have more questions?*

3. Lack of motivation: counseling

1. *Do your blood pressure lowering drug(s) bother you in any way?*

   If yes: Explore in what way the drug bother the patient. If it is related to the high concerns and low necessity, continue with the following questions.

2. *Do you think that it is important to use blood pressure lowering drug(s)?*
   a. If yes: *Can you explain this to me?*
   b. If no or insufficient: Explain that the blood pressure lowering drug(s) only works when taken as prescribed. If patients answer that they did not feel anything changed after taking the drug, explain that blood pressure

lowering drug(s) will not necessarily make them feel any different, but this does not mean the drug is not working. When relevant, also explain the importance of the blood pressure lowering drug(s) in reducing blood pressure level and prevent future cardiovascular diseases.

3. *Do you have any concerns when using blood pressure lowering drug(s)?*
    a. If no: Continue with the following question.
    b. If yes: Explore what the concerns are. If it is related to side effects, try to reduce it by explaining the common possible side effects which is not dangerous and by convincing patients that not everybody experienced the side effects. For example, start with this statement:
    Most people will be able to take their medicines without any problems. However, a few people can have side effects from their medicines. Everyone is different, and there is no way to tell whether you will have a side effect from your medicine. Most people who have side effects will feel them soon after they start to take a new medicine, or a higher dose of their medicine. (Of note, most of the more serious side effects of blood pressure lowering drug(s) are rare, for example, severe allergic reactions may affect up to 1 in 1,000 people or severe skin disorders -a sudden, unexpected rash or burning- may affect up to 1 in 10,000 people).

    Continue with this statement to reduce concerns about becoming too dependent on blood pressure lowering drug(s):

High blood pressure is a long-term problem that requires long-term treatment to keep it under control. For this reason, most people need to take tablets to keep their blood pressure down for the rest of their lives. However, a few people are able to bring their blood pressure down by really sticking to changes in their lifestyle, take the drug as prescribed, and later may be able to reduce the dose or some of their tablets.

4. *Are you worried about any side effects in the long term?*
   a. If no: Continue with the following question.
   b. If yes: Explain that some side effects may disappear over time, or patients may get used to them. Most side effects of blood pressure lowering drug(s) are not dangerous. Patients can always talk to doctor or pharmacist. For example*:*

      If you start to feel different after taking your blood pressure lowering drug(s) in the long-term, please talk to your doctor or to me. Your doctor may be able to change your dose, or try a different medicine.

4. Other problems: explore and offer possible solutions

1. *Do you have any side effects that you think may be caused by your blood pressure lowering drug(s)?*

   If yes: please call the Puskesmas or come over to Puskesmas.
2. *Do you have any other problem with taking or collecting your blood pressure lowering drug(s)?*
   a. If yes: Possible answers are due to experiencing side effect, polypharmacy, financial issue, lack of access to CHC, or the use of traditional medicine.

Patients may have other answers that are not listed here. Try to offer solutions according to their answer, for example, if patients have a difficulty to refill the drug in time, suggest them to ask for help from family of friend to accompany them to refill the blood pressure lowering drug(s).

b. If no: *Can you explain why you sometimes/often/always do not take your blood pressure lowering drug(s)?* (Some patients take less medication or lower dosages as 'self-management'; such patients may have good knowledge and motivation*)*

3. **Closing**

At the end of each counseling, involve patients in a goal setting process, for example to reduce forgetfulness. Ask their current blood pressure level and take it into account when discussing the goal. If patients already have a controlled blood pressure, explain that they can receive a reduced doses or reduced number of blood pressure lowering drug(s) (when relevant) in the future if they can maintain the blood pressure level under control. This must be done after discussing it first with the responsible doctor. After that, write the agreed goal at the top of the personalized leaflet for patients to take home. Please remind patients to take his/her leaflet in the next visit, and **make a copy of the leaflet for your own and the researchers to use**.

Follow-up session

1. **Review and discuss patient's implementation and experiences with the discussed plans and recommendations.**

1. For those who already adherent in the follow-up session: *You should be proud of all effort you have put into taking your blood pressure lowering drug(s) as prescribed to you.*
2. *What is your experience so far? How is it going?*

2. Identify all further barriers that were not yet addressed during the first session using the flowchart at 1-month follow up visit and provide the intervention based on the adherence intervention wheel.

Try to find out whether there are still any barriers for not using the blood pressure lowering drug(s). Ask more if something is not clear. Once any barriers have been identified, deliver the intervention accordingly (see baseline session).
