# Supplementary material for: Targeted and tailored pharmacist-led intervention to improve adherence to antihypertensive drugs among patients with type 2 diabetes in Indonesia: study protocol of a cluster randomised controlled trial
Source: BMJ Open. 2020 Jan 6;10(1):e034507. doi: 10.1136/bmjopen-2019-034507 (PMC6955569; doi:10.1136/bmjopen-2019-034507)
Supplement: Author's manuscript [file bmjopen-2019-034507.draft_revisions.pdf]

BMJ Open is committed to open peer review. As part of this commitment we make the peer review history of every article we publish publicly available.

When an article is published we post the peer reviewers' comments and the authors' responses online. We also post the versions of the paper that were used during peer review. These are the versions that the peer review comments apply to.

The versions of the paper that follow are the versions that were submitted during the peer review process. They are not the versions of record or the final published versions. They should not be cited or distributed as the published version of this manuscript.

BMJ Open is an open access journal and the full, final, typeset and author-corrected version of record of the manuscript is available on our site with no access controls, subscription charges or pay-per-view fees (http://bmjopen.bmj.com).

If you have any questions on BMJ Open's open peer review process please email info.bmjopen@bmj.com

BMJ Open

Targeted and tailored pharmacist-led intervention to improve adherence to antihypertensive drugs among patients with type 2 diabetes in Indonesia: design of a cluster randomized controlled trial

Journal:	*BMJ Open*
Manuscript ID	bmjopen-2019-034507
Article Type:	Protocol
Date Submitted by the Author:	24-Sep-2019
Complete List of Authors:	Alfian, Sofa; University of Groningen, ; Universitas Padjadjaran,<br>Abdulah, Rizky; Universitas Padjadjaran<br>Denig, Petra; University Medical Centre Groningen<br>van Boven, Job ; University Medical Centre Groningen<br>Hak, Eelko; University of Groningen
Keywords:	Diabetes & endocrinology < INTERNAL MEDICINE, medication adherence, intervention study

SCHOLARONE™
Manuscripts

Targeted and tailored pharmacist-led intervention to improve adherence to antihypertensive drugs among patients with type 2 diabetes in Indonesia: design of a cluster randomized controlled trial

Sofa D. Alfian [1,2,4*], Rizky Abdulah [2,4], Petra Denig [3], Job F. M. van Boven [3], Eelko Hak [1]

[1] University of Groningen, Groningen Research Institute of Pharmacy, Unit Pharmaco-Therapy, -Epidemiology & -Economics, Groningen, the Netherlands

[2] Universitas Padjadjaran, Faculty of Pharmacy, Department of Pharmacology and Clinical Pharmacy, Jatinangor, Indonesia

[3] University of Groningen, University Medical Centre Groningen, Department of Clinical Pharmacy and Pharmacology, Groningen, the Netherlands

[4] Universitas Padjadjaran, Center of Excellence in Higher Education for Pharmaceutical Care Innovation, Jatinangor, Indonesia

*Corresponding author: E-mail address: sofa.alfian@unpad.ac.id

For peer review only

For peer review only - http://bmjopen.bmj.com/site/about/guidelines.xhtml

ABSTRACT

Introduction: Current intervention programs to improve drug adherence are either too complex or expensive for implementation and scale-up in low-middle income countries. The aim of this study is to assess the process and effects of implementing a low-cost targeted and tailored pharmacist intervention among patients with type 2 diabetes who are non-adherent to antihypertensive drugs in a real-world primary care Indonesian setting.

Methods and analysis: A cluster randomized controlled trial with 3-month follow-up will be conducted in 10 community health centers (CHCs) in Indonesia. Type 2 diabetes patients aged 18 years and older who reported non-adherence to antihypertensive drugs according to the Medication Adherence Report Scale (MARS) are eligible to participate. Patients in CHCs randomized to the intervention group will receive a tailored intervention based on their personal adherence barriers. Interventions may include reminders, habit-based strategies, family support, counseling to educate and motivate patients, and strategies to address other drug-related problems. Interventions will be provided at baseline and at 1-month follow-up. Simple question-based flow-charts and an innovative adherence intervention wheel are provided to support the pharmacy staff. Patients in CHCs randomized to the control group will receive usual care based on the Indonesian guideline. Primary outcome is the between-group difference in medication adherence change from baseline to 3-month follow-up assessed by MARS. Secondary outcomes include changes in patients' blood pressure, their medication beliefs assessed by the Beliefs about Medicines Questionnaire (BMQ)-specific, as well as process characteristics of the intervention program from a pharmacist and patient perspective.

Ethics and dissemination: Ethical approval was obtained from the Ethical Committee of Universitas Padjadjaran, Indonesia and all patients will provide written informed consent prior to participation. The findings of the study will be disseminated through international conferences, one or more peer-reviewed journals, and reports to key stakeholders.

Trial registration: clinicaltrials.gov identifier: NCT04023734

For peer review only

For peer review only - http://bmjopen.bmj.com/site/about/guidelines.xhtml

Article Summary

Strengths and limitations of this study

- The pharmacist-led intervention program uses principles of targeting by screening for non-adherence and tailoring to the patients' personal adherence problems to enhance its potential effect. Simple question-based flow-charts and an innovative adherence intervention wheel are provided to support the pharmacy staff.
- The intervention aligns with the current workflow and resources in daily clinical practice of a lower-middle income country and will not require a substantial change to the current care system.
- This study is designed as a cluster randomized controlled trial to reduce risk of bias and contamination across study groups.
- The implementation process will be evaluated using the RE-AIM framework, which will enable us to carefully interpret the results and guide us when scaling up the intervention to include a larger population in Indonesia and other lower-middle income settings.
- As is common with many behavioral intervention studies, it is not possible to blind the researchers and pharmacists to the group allocation of patients.

For peer review only

INTRODUCTION

In patients with type 2 diabetes, pharmacological treatment of comorbid hypertension can substantially reduce the risk of cardiovascular complications.[1] However, while effective pharmacological treatment is available, adherence to antihypertensive medications in patients with type 2 diabetes is known to be suboptimal.[2] Notably, non-adherence to antihypertensive medications is associated with poor health outcomes and increased healthcare costs.[3] Therefore, effective intervention strategies to enhance adherence are urgently required.

Previous studies showed that patients may not take their medication for various reasons. Non-adherence could arise following a conscious decision after balancing the pros and cons of medication (intentional non-adherence), could be due to a lack of understanding the medication regimen, or due to forgetfulness (unintentional non-adherence).[4–7] The reasons underlying intentional and unintentional non-adherence are not entirely independent, and are heterogeneous. These reasons include lack of attention, lack of knowledge, high concerns and/or low necessity beliefs, which can reduce motivation.[4–7] In addition, there may be other drug-related problems, such as difficulties with intake or high costs, that can lead to non-adherence.[8,9] As such, there are no one-size-fits-all solutions to address non-adherence.

In developed countries with well-established healthcare systems, a wide variety of interventions to improve medication adherence have been developed.[10,11] Six main types of interventions can be identified: patient education, medication regimen management, clinical pharmacist consultation, cognitive behavioral therapies, medication-taking reminders, and incentives to promote adherence.[11] However, a Cochrane review showed that most interventions are often too complex and not particularly effective.[10] Additionally, in lower-middle income countries,

For peer review only - http://bmjopen.bmj.com/site/about/guidelines.xhtml

the paucity of healthcare and economic resources poses challenges to proper implementation of adherence enhancing interventions.

Non-adherence may be more efficiently improved if only patients who need it are targeted and interventions are tailored to patients' individual adherence barriers.[12,13] Previous studies further suggested that effective interventions to improve adherence were led by a pharmacist, delivered face-to-face, administered directly to patients, and behaviourally-targeted compared to cognitively-targeted interventions.[14] In Southeast Asia, however, pharmacy services can be hampered by inadequate training of pharmacy staff.[15] Furthermore, adherence interventions may not be sustained over time due to a lack of resources to maintain them. Thus, to improve adherence to antihypertensive drugs in patients with type 2 diabetes, a low-cost, targeted and tailored pharmacist-led intervention that can be integrated into the community pharmacy workflow is required.

The primary objective of this study is to assess the effect of a targeted and tailored pharmacist-led intervention on medication adherence to antihypertensive drugs among type 2 diabetes patients. The secondary objectives are to assess the effect of the intervention on blood pressure level and medication beliefs, and to evaluate the implementation of the intervention from a pharmacist and patient perspective.

For peer review only

METHODS AND ANALYSIS

This protocol was developed in accordance with the CONSORT 2010 statement for cluster randomized trials[16] and reported according to the SPIRIT checklist[17] (Supplementary appendix A). We will use the RE-AIM framework[18] to evaluate the implementation process of the intervention.

Study design and setting

This study is a 3-month cluster randomized controlled trial with two parallel arms and will be performed in Bandung City, Indonesia from August to December 2019 (Figure 1). Clusters of randomization are community health centres (CHCs), locally called *Puskesmas*. *Puskemas* are primary health care centres at the sub-district level, with each centre staffed with medical doctors, nurses, midwives, and pharmacists.

Participants

A total of 10 CHCs will be purposively selected based on a sufficient number of type 2 diabetes patients with hypertension. The principle investigator (PI) will introduce and explain the study to the pharmacists and physicians in the CHCs. In each CHC, one pharmacist will be included. In Indonesia, one of the pharmacist's responsibilities during routine clinical practice is to counsel patients with chronic diseases on their medication use, often performed in a counselling room which is separated from the drug counter.[19] Screening for patients' eligibility will be conducted by the pharmacist during regular outpatient visits. Patients will be eligible if they meet the following inclusion criteria: (i) at least 18 years old, (ii) diagnosed with type 2 diabetes for at least one year based on patient's medical record, (iii) using at least one antihypertensive drug in the last three months. Patients with severe mental or physical constraints, pregnancy or

For peer review only - http://bmjopen.bmj.com/site/about/guidelines.xhtml

in the lactation period, illiterate in the Indonesian language, enrolment in another intervention study, and those not responsible for taking their own medication will be excluded. Once a patient is deemed eligible, the pharmacist will inform the PI or research assistant to approach the patient and briefly explain the study, and ask to sign informed consent. Eligible patients who have signed informed consent will be screened for suboptimal medication adherence to antihypertensive drugs according to the Medication Adherence Report Scale (MARS score <20; MARS scores range from 5 to 25) before patients go to the counselling room. Of note, our previous work in four regions in Indonesia (Bandung City, Yogyakarta City, Makassar City, and Samarinda city) showed that half of patients were non-adherent with a MARS score < 20.

Randomization and blinding

We will use cluster randomization at the CHCs level to reduce risk of bias and contamination across study groups.[20] The PI will randomize the CHCs into the intervention or control group in a 1:1 ratio. The randomization sequence will be generated using a random number generator. Given the nature of the study design, both pharmacists and the PI cannot be blinded to the group assignment.

Intervention

Patients in the five CHCs randomized to the intervention group who were screened as non-adherent to their antihypertensive drugs will receive a tailored pharmacist-led intervention during two sessions (at baseline and at 1-month follow-up) in addition to usual care. Both will be regular outpatient visits, when patients collect their medication. The intervention will be low-cost, align with the current CHC workflow and will not require a substantial change to the

For peer review only - http://bmjopen.bmj.com/site/about/guidelines.xhtml

For peer review only

current system. Before the study started, the intervention steps and materials were piloted in two pharmacies and optimized in an iterative process.

Intervention at baseline (first session)

Before dispensing antihypertensive drugs during the first session, the pharmacist will discuss patient-specific barrier(s) for medication adherence based on their responses to the MARS questionnaire and three additional questions, which are derived from the Brief Medication Questionnaire[21] (Figure 2). The intervention strategy will then be tailored to the identified adherence problems. Based on current literature[22–24], we defined four non-adherence problems that can be addressed by the community pharmacists, i.e. (1) forgetfulness, (2) lack of knowledge, (3) lack of motivation and/or (4) other drug-related problems. Of note, patients might need a combined intervention strategy to address all experienced problems. The four non-adherence problems and recommended intervention strategies are specified in Table 1. The session will end with involving patients in goal setting and writing the agreed goal at the top of the personalized leaflet. Pharmacists will remind patients to take his/her leaflet to the next visit.

Table 1 Non-adherence problems and recommended intervention strategies

Non-adherence problems	Intervention strategies
1. Forgetfulness	Strategies to cope with forgetfulness include reminders, habit-based strategies and/or involvement of family members, and improving knowledge on what to do when a dose is forgotten.[23] The use of a reminder tool or pill boxes will be encouraged and a reminder app can be implemented if the patient owns a mobile phone. The habit-based strategy will be delivered through a personalized leaflet, which is tailored to patient's daily routine (Supplementary appendix B). Patients

For peer review only - http://bmjopen.bmj.com/site/about/guidelines.xhtml

	will be asked to identify the appropriate place and time to take their medication, and an activity they conduct every day that could serve as a prompt or cue to take their medication.[25] Patients will be asked to write coping plans to formulate their own "if–then" plans for the daily doses of their antihypertensive drug(s).[25] Moreover, patients will be asked to choose a family member to become their treatment supporter and to write down the name of family member on the personalized leaflet. This individual will be asked to support the patient to take the antihypertensive drugs. Pharmacists will keep a copy of the leaflet and remind patients to take his/her personalized leaflet to the next visit.
2. Lack of knowledge	Patient counseling by the pharmacist to cope with lack of knowledge may focus on educating the patient about the purpose of the medication, when and how to take the medication, the need for long-term use, the importance of medication adherence, and how to deal with possible side effects. To explore which education is needed, the patient will be asked whether they know why and how to take their medication. The teach-back method will be used, where the patient is asked to explain the pharmacist what he/she has understood after receiving the education.[26]
3. Lack of motivation	Counseling to cope with lack of motivation will focus on exploring and discussing the patients' concerns and necessity beliefs. This method is called motivational interviewing.[27] This is done by asking a first question about whether the medication bothers the patient. Follow-up on this question can focus on reducing any concerns or low necessity beliefs (e.g., when patients are bothered by the medication because they think the medication is not needed or are afraid of side-effects).
4. Other drug-related problems	Counseling to address other drug-related problems will focus on exploring other problems underlying non-adherence, for example, experiencing side effects, costs, polypharmacy, difficulty to refill antihypertensive drugs in time, or medication intake problems, and offering solutions/alternatives when possible.

For peer review only

Interventions at follow-up (second session)

The follow-up session will be conducted one month after the baseline session, when patients refill their medication at the next regular outpatient visit (Figure 3). The purpose of the follow-up session is (1) to evaluate the short-term effect of the intervention and discuss the patients' implementation of and experiences with the offered information and recommendations, and (2) to address non-adherence problems that were not yet addressed during the first session. Where needed, the pharmacist, together with patient, can make changes to the coping plan and discuss additional interventions. The session will end with involving patients in goal setting and writing the agreed goal at the top of the personalized leaflet.

Pharmacist training

As the quality of the intervention will depend on the competences and skills of the pharmacist, treatment integrity will be enhanced by an obligatory communication training focusing on how to elicit and classify barriers to adherence, the teach-back method and motivational interviewing, and by providing supportive material as part of the intervention (Figure 4).

Pharmacists and patients participating in this study will be compensated with a modest souvenir at the end of the study for their time and effort.

Control group

Patients in the five CHCs randomized to the control group will receive pharmacist counseling based on the Indonesian guideline of pharmacy practice (PMK No.74/2016).[19] At each visit, they can receive information about the quantity and dose of the dispensed drugs, when and how to use and store the drugs, side effects and how to deal with them, the importance of medication adherence, and confirming if the patient understands how to take medications correctly. Patients

For peer review only - http://bmjopen.bmj.com/site/about/guidelines.xhtml

For peer review only

in the control group who were screened as non-adherent to their antihypertensive drugs by the research assistant at baseline will complete the assessments at the same time points as those in the intervention group.

Outcomes

Primary outcome

The primary outcome is the difference between the intervention and the control group in change in MARS score from baseline (T0) to 3-month follow-up (T2). The Indonesian version of the MARS showed to be valid (correlation value of each question to the total score > 0.396) and reliable (Cronbach α coefficient of 0.803).[28] Patients will indicate how often each statement applied to them in the last three months on a 5-point Likert scale ranging from always (score 1) to never (score 5). Items are summed to obtain a total score ranging from 5 to 25 (Horne R. The Medication Adherence Report Scale (MARS): a new measurement tool for eliciting patients' reports of non-adherence).

Secondary outcomes

1. Blood pressure level

   Within and between patient changes in blood pressure (BP) level (systolic blood pressure [SBP] and diastolic blood pressure [DBP]) will be assessed. BP measurements will be performed by a nurse who is blinded to the group assignment at baseline (T0), 1-month (T1), and 3-month follow-up (T2).

2. Medication beliefs

   Within patient changes on beliefs about medication will be assessed using the Beliefs about Medicines Questionnaire (BMQ)-specific at baseline (T0) and 3-month follow-up (T2). The Indonesian version of the BMQ-specific showed to be valid (correlation value

For peer review only - http://bmjopen.bmj.com/site/about/guidelines.xhtml

For peer review only

of each question to the total score > 0.530) and reliable (Cronbach α coefficient of 0.835 and 0.811 for necessity and concerns beliefs, respectively). The BMQ-specific contains five items about necessity beliefs (e.g. "My health at present depends on my blood pressure-lowering medicines"), five items about concern beliefs (e.g. "I sometimes worry about becoming too dependent on my blood pressure-lowering medicines"), and one item about side effects (e.g. 'My blood pressure-lowering medicines gives me unpleasant side effects). All items have a 5-point Likert scale ranging from strongly disagree to strongly agree with an overall range from 5 (low necessity, low concern) to 25 (high necessity, high concern). A necessity–concern differential score will be calculated by subtracting the scores of the concerns scale from the necessity scale (range −20 to 20). A positive differential score indicates stronger beliefs in the necessity, while a negative score indicates stronger concerns.[29] The additional item about side effects will be analyzed separately due to its known role in non-adherence.[22,30]

3. Process evaluation

We will conduct a process evaluation to assess other parts of the RE-AIM framework. In short, the RE-AIM framework has been developed to evaluate public health interventions assessing five dimensions (reach, efficacy, adoption, implementation and maintenance) at multiple levels (e.g., individual or organization).[18] *Reach* will be assessed by measuring the participation rates and representativeness of patients who participate in this study. In case a patient refuses or discontinues to participate in this study, the patient's age, gender, and BP lowering drugs the patient uses will be recorded by research assistants. This information is used to calculate the participation rate and assess differences between responders and non-responders. To determine representativeness, the patients' demographics will be compared to census demographics in Bandung City, Indonesia.

For peer review only - http://bmjopen.bmj.com/site/about/guidelines.xhtml

Adoption will be evaluated by assessing the proportion and representativeness of CHCs who participate in this study, and exploring pharmacists' and patients' satisfaction with and willingness to use various parts of the intervention. *Implementation* will be evaluated by determining whether the intervention was delivered as intended and exploring pharmacists' and patients' suggestions for future implementation. *Maintenance* will be assessed by determining whether the intervention can be maintained and the willingness of pharmacists and payers to continue the intervention as part of routine clinical practice. We will use focus group discussions and an evaluation survey (based on a previously used survey[31]) to explore pharmacists' and patients' adoption, implementation, and willingness to maintain the intervention, respectively.

Baseline participant and CHC characteristics

Participants' baseline characteristics, including sociodemographic and clinical-related factors, will be obtained. Sociodemographic factors are self-reported and include age at the completion of the questionnaire, gender, highest level of education completed (no formal education/elementary high school, junior high school, senior high school, or university), and type of health insurance. Type of health insurance will be classified as those whose insurance premium was paid by the government (BPJS-PBI), those whose insurance premium was paid by the patients themselves (BPJS-Non PBI), or those without health insurance. Clinical-related factors include time since diagnosis of diabetes and hypertension (years), diabetes complication(s) that developed after the diagnosis of diabetes, and types and number of concomitant medications. The following diabetes complications will be considered: cardiovascular conditions, cerebrovascular conditions, nephropathy, retinopathy, neuropathy, and diabetic foot problems. Clinical-related factors will be collected by research assistants using a predefined data collection form. Furthermore, organizational information of each CHC

For peer review only - http://bmjopen.bmj.com/site/about/guidelines.xhtml

(number of medical doctors, pharmacists, nurses, and average number of diabetes patients with and without hypertension visits per month) and pharmacist characteristics (age, gender, and working experience in community pharmacy [years]) will be collected by research assistants using a predefined data collection form.

Treatment fidelity

Treatment fidelity will be addressed by providing a checklist of items that pharmacists need to do at each patient visit and a counselling protocol for the intervention group (Supplementary appendix C). Pharmacists will be asked to complete the checklist after each visit with a study participant. The completed checklists will be collected on a weekly basis and used to calculate an overall fidelity score. Minor feedback suggestions from the PI to pharmacists will be made if needed.

Sample size calculation

The sample size calculation is based on the formula for cluster randomized trials, powered on the primary outcome.[32] We want to be able to detect a difference between the intervention and control group in change in adherence score of at least 2.5 points with an expected standard deviation of 3.8 points and assuming an intra-cluster correlation coefficient within CHCs of 0.014, as calculated based on previous work from our group (manuscript in preparation). A sample size of 41 non-adherent patients in each study arm (intervention and control group) would allow for 80% power to detect this difference using a two-sided test at the 5% level of significance. Assuming non-adherence rates of 50%[33] and a dropout rate of 20%, we will recruit at least 100 patients in each group, giving a total of 200 patients from 10 CHCs (20 per CHC) that need to complete the MARS screening. Recruiting at least 20 patients per CHC is feasible

For peer review only - http://bmjopen.bmj.com/site/about/guidelines.xhtml

since the average number of diabetes patients with hypertension visiting a CHC is around 30 patients per month.

Planned statistical analysis

Data analysis will be performed based on the intention-to-treat principle. Descriptive statistics will be used to summarize the baseline characteristics. To control for the effects of cluster randomization, group changes will be compared (individual CHCs will be treated as a random effect) using multivariate mixed linear and nonlinear regression for normally and non-normally distributed data, respectively. Point estimates estimated from cluster-adjusted models will be reported with 95% confidence intervals. All tests will be two-tailed and $p<0.05$ will be considered statistically significant. All statistical analyses will be carried out using SPSS software (version 25.0; IBM, Armonk, NY, USA).

Data management

Data from the questionnaires and case report forms will be entered by the PI using a unique identifier that is provided for each participant into SPSS software version 25 and data forms will be stored on a password-protected computer.

Adverse event reporting

It is possible that a participant identifies a medication-related issue during pharmacist counseling, either in the intervention or control group. Although this is unlikely to be a result from the study, the patient's doctor will be contacted if the pharmacist and/or researchers have concerns requiring immediate intervention.

For peer review only

Patient and public involvement

Patients and public were not involved in the development of the research question or outcome measures. Patients will be involved during the conduct of the study by giving feedback to tailor the intervention based on their personal adherence barrier(s). In addition, patients will be asked to complete an evaluation survey, including questions about the intervention. Patients will be given contact details of the PI to request the results of the study.

For peer review only

For peer review only - http://bmjopen.bmj.com/site/about/guidelines.xhtml

ETHICS AND DISSEMINATION

Ethical approval for this study was obtained from the Ethical Committee of Universitas Padjadjaran, Indonesia (No. 859/UN6.KEP/EC/2019). All results will be stored securely and will be available to authorized individuals for analysis and reporting purposes only. Data will be published in a form that does not identify patients in any way. To maintain patients' anonymity, a unique identifier will be used to match patients' data across baseline and follow-up. Patients are free to withdraw from the study at any time. The findings of the study will be disseminated through international conferences, one or more peer-reviewed journals, and reports to key stakeholders.

Authors' contributions

SDA wrote the first draft of this protocol. SDA, RA, JFMvB, PD, and EH participated in the design of the study and contributed to the revision of the study protocol. All authors approved the final manuscript.

Funding

SDA is supported by a scholarship from the Indonesia Endowment Fund for Education (LPDP No: PRJ-2361/LPDP/2015). This funding body did not have any role in designing the study, in writing this article, and in deciding to submit it for publication.

Competing interests

None declared.

For peer review only

REFERENCES

1. American Diabetes Association. 10. Cardiovascular Disease and Risk Management: *Standards of Medical Care in Diabetes—2019*. Diabetes Care. 2019;42(Supplement 1):S103-23.

2. Ledur PS, Leiria LF, Severo MD, et al. Perception of uncontrolled blood pressure and non-adherence to anti-hypertensive agents in diabetic hypertensive patients. J Am Soc Hypertens. 2013;7(6):477-83.

3. Khan R, Socha-Dietrich K. Investing in medication adherence improves health outcomes and health system efficiency: adherence to medicines for diabetes, hypertension, and hyperlipidaemia. OECD Heal Work Pap. 2018;(105).

4. Rees G, Leong O, Crowston JG, Lamoureux EL. Intentional and unintentional nonadherence to ocular hypotensive treatment in patients with glaucoma. Ophthalmology. 2010;117(5):903-8.

5. Iihara N, Kurosaki Y, Miyoshi C, Takabatake K, Morita S, Hori K. Comparison of individual perceptions of medication costs and benefits between intentional and unintentional medication non-adherence among Japanese patients. Patient Educ Couns. 2008;70(2):292-9.

6. Wroe AL. Intentional and unintentional nonadherence: a study of decision making. J Behav Med. 2002;25(4):355-72.

7. Clifford S, Barber N, Horne R. Understanding different beliefs held by adherers, unintentional nonadherers, and intentional nonadherers: Application of the Necessity-Concerns Framework. J Psychosom Res. 2008;64(1):41-6.

8. Sabate E. *Adherence to Long-Term Therapies: Evidence for Action*. World Health Organization; 2003.

9. Mathes T, Jaschinski T, Pieper D. Adherence influencing factors - a systematic review of systematic reviews. Arch Public Heal. 2014;72(1):1-9.

10. Nieuwlaat R, Wilczynski N, Navarro T, et al. Interventions for enhancing medication adherence. Cochrane Database Syst Rev. 2014;(11).

11. Kini V, Ho PM. Interventions to improve medication adherence. JAMA. 2018;320(23):2461.

12. Cutrona SL, Choudhry NK, Fischer MA, et al. Targeting cardiovascular medication adherence interventions. J Am Pharm Assoc. 2012;52(3):381-97.

13. van Boven JFM, Stuurman-Bieze AGG, Hiddink EG, Postma MJ, Vegter S. Medication Monitoring and Optimization: A Targeted Pharmacist Program for Effective and Cost-Effective Improvement of Chronic Therapy Adherence. J Manag Care Pharm. 2014;20(8):786-92.

14. Conn VS, Ruppar TM. Medication adherence outcomes of 771 intervention trials: Systematic review and meta-analysis. Prev Med (Baltim). 2017;99:269-76.

15. Hermansyah A, Sainsbury E, Krass I. Community pharmacy and emerging public

For peer review only

health initiatives in developing Southeast Asian countries: a systematic review. Health Soc Care Community. 2016;24(5):e11-22.

16. Campbell MK, Piaggio G, Elbourne DR, Altman DG. Consort 2010 statement: Extension to cluster randomised trials. BMJ. 2012;345(7881):1-21.

17. Chan A-W, Tetzlaff JM, Gøtzsche PC, et al. SPIRIT 2013 explanation and elaboration: guidance for protocols of clinical trials. BMJ. 2013;346:e7586.

18. Glasgow RE, Vogt TM, Boles SM. Evaluating the public health impact of health promotion interventions: the RE-AIM framework. Am J Public Health. 1999;89(9):1322-7.

19. Indonesia KKR. *Peraturan Menteri Kesehatan Republik Indonesia Nomor 74 Tahun 2016 Tentang Standar Pelayanan Kefarmasian Di Puskesmas*. Jakarta; 2016. http://jdih.pom.go.id/produk/peraturan menteri/PMK_No._74_ttg_Standar_Pelayanan_Kefarmasian_di_PUSKESMAS_.pdf. Accessed February 22, 2019.

20. Puffer S, Torgerson DJ, Watson J. Cluster randomized controlled trials. J Eval Clin Pract. 2005;11(5):479-83.

21. Svarstad BL, Chewning BA, Sleath BL, Claesson C. The Brief Medication Questionnaire: a tool for screening patient adherence and barriers to adherence. Patient Educ Couns. 1999;37(2):113-24. http://www.ncbi.nlm.nih.gov/pubmed/14528539. Accessed April 17, 2018.

22. van der Laan DM, Elders PJM, Boons CCLM, Beckeringh JJ, Nijpels G, Hugtenburg JG. Factors associated with antihypertensive medication non-adherence: a systematic review. J Hum Hypertens. 2017;31(11):687-94.

23. van der Laan DM, Elders PJM, Boons CCLM, Nijpels G, Hugtenburg JG. Factors associated with nonadherence to cardiovascular medications. J Cardiovasc Nurs. 2019;34(4):344-52.

24. van Boven JFM, Lavorini F, Dekhuijzen PNR, Blasi F, Price DB, Viegi G. Urging Europe to put non-adherence to inhaled respiratory medication higher on the policy agenda: a report from the First European Congress on Adherence to Therapy. Eur Respir J. 2017;49(5).

25. Meslot C, Gauchet A, Hagger MS, Chatzisarantis N, Lehmann A, Allenet B. A randomised controlled trial to test the effectiveness of planning strategies to improve medication adherence in patients with cardiovascular disease. Appl Psychol Heal Well-Being. 2017;9(1):106-29.

26. Dinh HTT, Bonner A, Clark R, Ramsbotham J, Hines S. The effectiveness of the teach-back method on adherence and self-management in health education for people with chronic disease: a systematic review. JBI Database Syst Rev Implement Reports. 2016;14(1):210.

27. Rollnick S, Miller WR, Arkowitz H, Westra HA, Butler CC. *Motivational Interviewing in Health Care: Helping Patients Change Behavior*. New York: The Guilford Press; 2008.

For peer review only

For peer review only - http://bmjopen.bmj.com/site/about/guidelines.xhtml

28. Alfian R, Putra AMP. Uji validitas dan reliabilitas kuesioner medication adherence report scale (MARS) terhadap pasien diabetes mellitus [in Bahasa Indonesia]. J Ilm Ibnu Sina. 2017;2:176-83.

29. Horne R, Weinman J, Hankins M. The beliefs about medicines questionnaire: The development and evaluation of a new method for assessing the cognitive representation of medication. Psychol Heal. 1999;14(1):1-24.

30. Birtcher K. When compliance is an issue—how to enhance statin adherence and address adverse effects. Curr Atheroscler Rep. 2015;17(1). https://link.springer.com/content/pdf/10.1007%2Fs11883-014-0471-8.pdf. Accessed February 22, 2019.

31. Stuurman-Bieze AGG, Hiddink EG, van Boven JFM, Vegter S. Proactive Pharmaceutical Care Interventions Improve Patients' Adherence to Lipid-Lowering Medication. Ann Pharmacother. 2013;47(11):1448-56.

32. Rutterford C, Copas A, Eldridge S. Methods for sample size determination in cluster randomized trials. Int J Epidemiol. 2015;44(3):1051-67.

33. Adikusuma W, Perwitasari DA, Supadmi W. Evaluasi kepatuhan pasien diabetes melitus tipe 2 di Rumah Sakit Umum PKU Muhammadiyah Bantul, Yogyakarta [in Bahasa Indonesia]. Media Farm. 2014;11(2):208-20. http://journal.uad.ac.id/index.php/Media-Farmasi/article/view/1880. Accessed July 12, 2018.

For peer review only

For peer review only - http://bmjopen.bmj.com/site/about/guidelines.xhtml

Figures

Figure 1 Consort flow chart

Abbreviation: BP: Blood pressure; CHC: Community health centre; MARS = Medication adherence report scale; BMQ-specific = Beliefs about medicines questionnaire-specific; FGD: Focus group discussions

Figure 2 Flow chart of a targeted and tailored intervention at baseline visit (T0)

Type of intervention: 1 = Reminders, habit-based strategies and/or involvement of family member; 2 = Counseling to increase knowledge (teach-back method); 3 = Counseling to increase motivation; 4 = Explore/address other drug related problems
Abbreviation: MARS: Medication adherence report scale

Figure 3 Flow chart of a targeted and tailored intervention at the one-month follow up visit (T1)

Type of intervention: 2 = Counseling to increase knowledge (teach-back method); 3 = Counseling to increase motivation; 4 = Explore/address other drug related problems
Abbreviation: MARS: Medication adherence report scale

Figure 4 Proposed adherence intervention wheel as supportive material for pharmacists in the intervention group

For peer review only

For peer review only - http://bmjopen.bmj.com/site/about/guidelines.xhtml

For peer review only

For peer review only - http://bmjopen.bmj.com/site/about/guidelines.xhtml

For peer review only

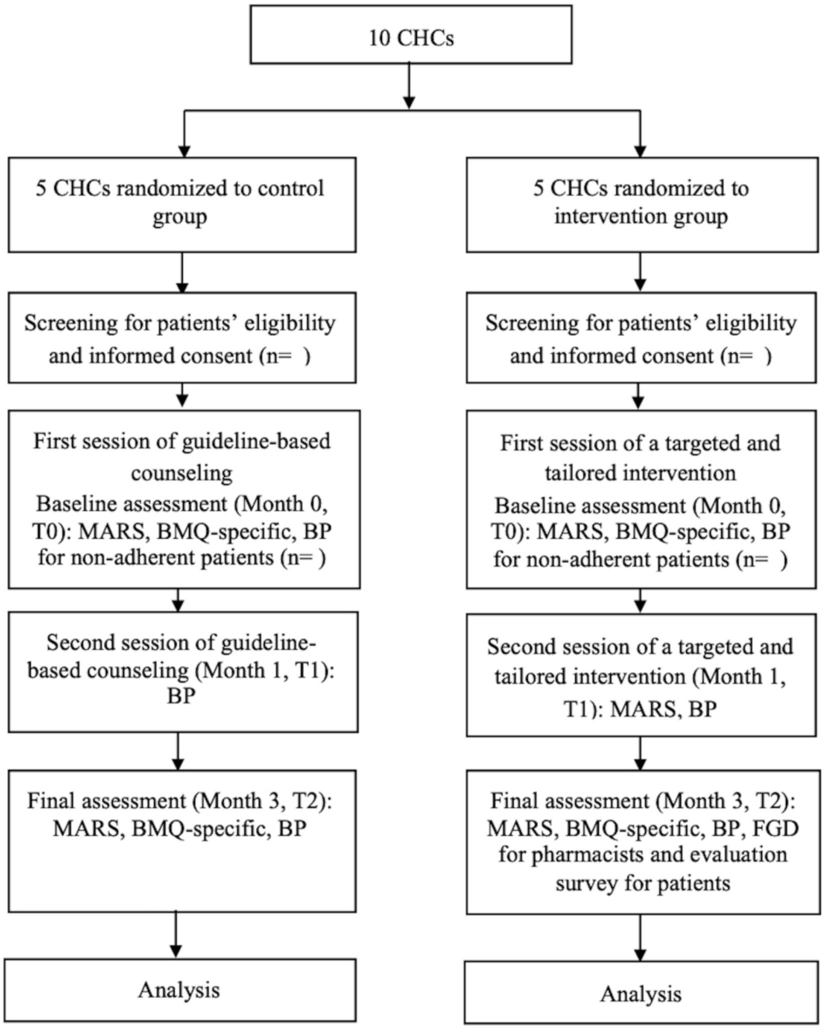

Figure 1 Consort flow chart
Abbreviation: BP: Blood pressure; CHC: Community health centre; MARS = Medication adherence report scale; BMQ-specific = Beliefs about medicines questionnaire-specific; FGD: Focus group discussions

164x187mm (300 x 300 DPI)

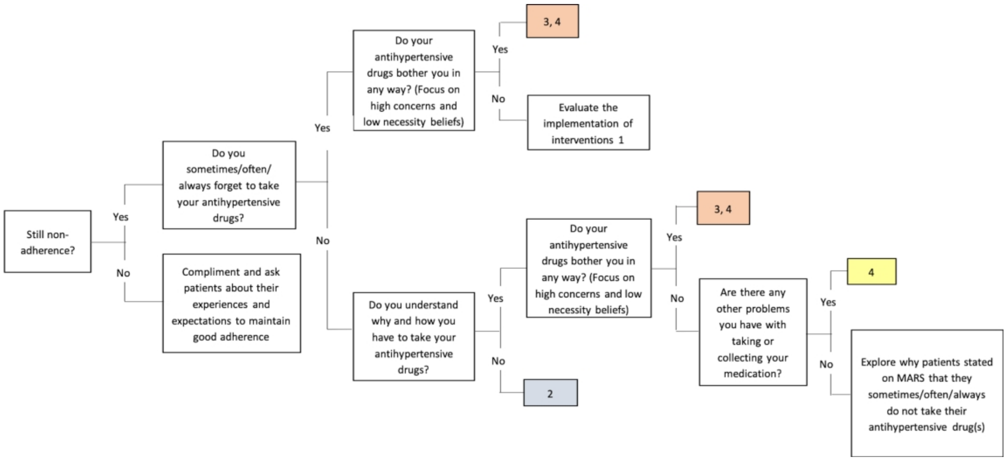

Figure 2 Flow chart of a targeted and tailored intervention at baseline visit (T0)
Type of intervention: 1 = Reminders, habit-based strategies and/or involvement of family member; 2 = Counseling to increase knowledge (teach-back method); 3 = Counseling to increase motivation; 4 = Explore/address other drug related problems
Abbreviation: MARS: Medication adherence report scale

307x139mm (300 x 300 DPI)

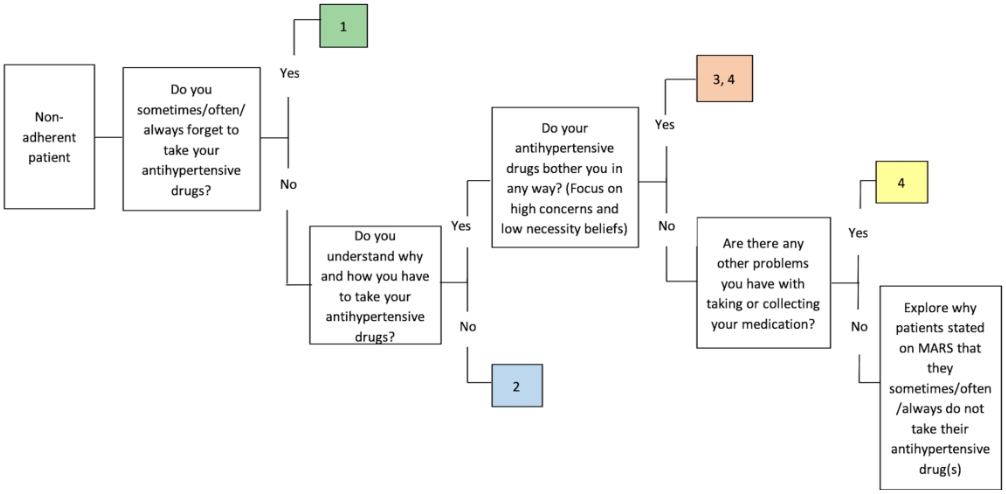

Figure 3 Flow chart of a targeted and tailored intervention at the one-month follow up visit (T1)
Type of intervention: 2 = Counseling to increase knowledge (teach-back method); 3 = Counseling to increase motivation; 4 = Explore/address other drug related problems
Abbreviation: MARS: Medication adherence report scale

289x140mm (300 x 300 DPI)

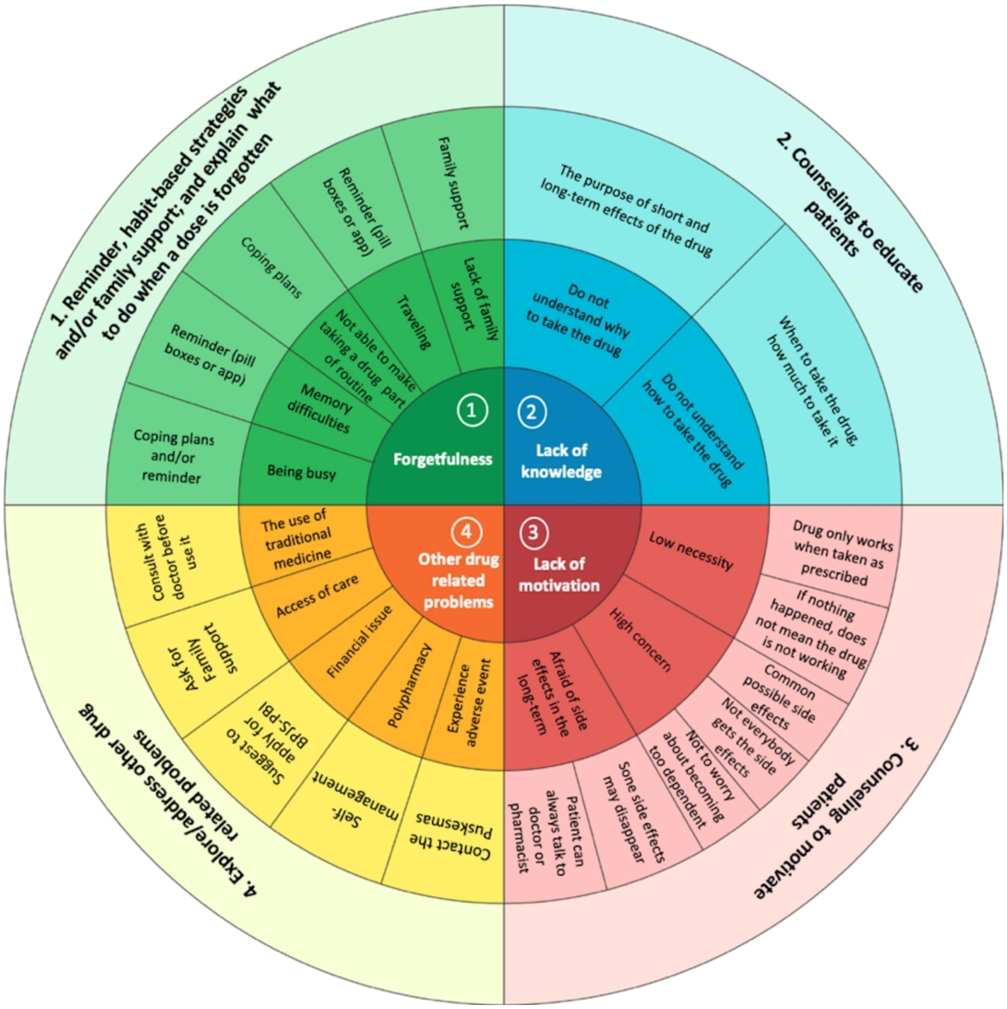

Figure 4 Proposed adherence intervention wheel as supportive material for pharmacists in the intervention group

159x159mm (300 x 300 DPI)

APPENDIX

A. SPIRIT checklist

SPIRIT
STANDARD PROTOCOL ITEMS: RECOMMENDATIONS FOR INTERVENTIONAL TRIALS

Section/item	Item No	Description	Reported on page
**Administrative information**			
Title	1	Descriptive title identifying the study design, population, interventions, and, if applicable, trial acronym	1
Trial registration	2a	Trial identifier and registry name. If not yet registered, name of intended registry	2
	2b	All items from the World Health Organization Trial Registration Data Set	4-20
Protocol version	3	Date and version identifier	NA
Funding	4	Sources and types of financial, material, and other support	17
Roles and responsibilities	5a	Names, affiliations, and roles of protocol contributors	1, 17
	5b	Name and contact information for the trial sponsor	NA
	5c	Role of study sponsor and funders, if any, in study design; collection, management, analysis, and interpretation of data; writing of the report; and the decision to submit the report for publication, including whether they will have ultimate authority over any of these activities	17
	5d	Composition, roles, and responsibilities of the coordinating centre, steering committee, endpoint adjudication committee, data management team, and other individuals or groups overseeing the trial, if applicable (see Item 21a for data monitoring committee)	NA

For peer review only - http://bmjopen.bmj.com/site/about/guidelines.xhtml

**Introduction**			
Background and rationale	6a	Description of research question and justification for undertaking the trial, including summary of relevant studies (published and unpublished) examining benefits and harms for each intervention	4-5
	6b	Explanation for choice of comparators	10
Objectives	7	Specific objectives or hypotheses	5
Trial design	8	Description of trial design including type of trial (eg, parallel group, crossover, factorial, single group), allocation ratio, and framework (eg, superiority, equivalence, non-inferiority, exploratory)	6,7
**Methods: Participants, interventions, and outcomes**			
Study setting	9	Description of study settings (eg, community clinic, academic hospital) and list of countries where data will be collected. Reference to where list of study sites can be obtained	6
Eligibility criteria	10	Inclusion and exclusion criteria for participants. If applicable, eligibility criteria for study centres and individuals who will perform the interventions (eg, surgeons, psychotherapists)	6,7
Interventions	11a	Interventions for each group with sufficient detail to allow replication, including how and when they will be administered	7-10
	11b	Criteria for discontinuing or modifying allocated interventions for a given trial participant (eg, drug dose change in response to harms, participant request, or improving/worsening disease)	NA
	11c	Strategies to improve adherence to intervention protocols, and any procedures for monitoring adherence (eg, drug tablet return, laboratory tests)	14
	11d	Relevant concomitant care and interventions that are permitted or prohibited during the trial	7
Outcomes	12	Primary, secondary, and other outcomes, including the specific measurement variable (eg, systolic blood pressure), analysis metric (eg, change from baseline, final value, time to event), method of aggregation (eg, median, proportion), and time point for each outcome. Explanation of the clinical relevance of chosen efficacy and harm outcomes is strongly recommended	11-13
Participant timeline	13	Time schedule of enrolment, interventions (including any run-ins and washouts), assessments, and visits for participants. A schematic diagram is highly recommended (see Figure)	Figure 1

For peer review only - http://bmjopen.bmj.com/site/about/guidelines.xhtml

Sample size	14	Estimated number of participants needed to achieve study objectives and how it was determined, including clinical and statistical assumptions supporting any sample size calculations	14
Recruitment	15	Strategies for achieving adequate participant enrolment to reach target sample size	14
**Methods: Assignment of interventions (for controlled trials)**			
Allocation:			
Sequence generation	16a	Method of generating the allocation sequence (eg, computer-generated random numbers), and list of any factors for stratification. To reduce predictability of a random sequence, details of any planned restriction (eg, blocking) should be provided in a separate document that is unavailable to those who enrol participants or assign interventions	7
Allocation concealment mechanism	16b	Mechanism of implementing the allocation sequence (eg, central telephone; sequentially numbered, opaque, sealed envelopes), describing any steps to conceal the sequence until interventions are assigned	7
Implementation	16c	Who will generate the allocation sequence, who will enrol participants, and who will assign participants to interventions	7
Blinding (masking)	17a	Who will be blinded after assignment to interventions (eg, trial participants, care providers, outcome assessors, data analysts), and how	7
	17b	If blinded, circumstances under which unblinding is permissible, and procedure for revealing a participant's allocated intervention during the trial	NA
**Methods: Data collection, management, and analysis**			
Data collection methods	18a	Plans for assessment and collection of outcome, baseline, and other trial data, including any related processes to promote data quality (eg, duplicate measurements, training of assessors) and a description of study instruments (eg, questionnaires, laboratory tests) along with their reliability and validity, if known. Reference to where data collection forms can be found, if not in the protocol	11-14
	18b	Plans to promote participant retention and complete follow-up, including list of any outcome data to be collected for participants who discontinue or deviate from intervention protocols	12, 14
Data management	19	Plans for data entry, coding, security, and storage, including any related processes to promote data quality (eg, double data entry; range checks for data values). Reference to where details of data management procedures can be found, if not in the protocol	15

Statistical methods	20a	Statistical methods for analysing primary and secondary outcomes. Reference to where other details of the statistical analysis plan can be found, if not in the protocol	14
	20b	Methods for any additional analyses (eg, subgroup and adjusted analyses)	NA
	20c	Definition of analysis population relating to protocol non-adherence (eg, as randomised analysis), and any statistical methods to handle missing data (eg, multiple imputation)	14
**Methods: Monitoring**			
Data monitoring	21a	Composition of data monitoring committee (DMC); summary of its role and reporting structure; statement of whether it is independent from the sponsor and competing interests; and reference to where further details about its charter can be found, if not in the protocol. Alternatively, an explanation of why a DMC is not needed	NA
	21b	Description of any interim analyses and stopping guidelines, including who will have access to these interim results and make the final decision to terminate the trial	NA
Harms	22	Plans for collecting, assessing, reporting, and managing solicited and spontaneously reported adverse events and other unintended effects of trial interventions or trial conduct	15
Auditing	23	Frequency and procedures for auditing trial conduct, if any, and whether the process will be independent from investigators and the sponsor	NA
**Ethics and dissemination**			
Research ethics approval	24	Plans for seeking research ethics committee/institutional review board (REC/IRB) approval	17
Protocol amendments	25	Plans for communicating important protocol modifications (eg, changes to eligibility criteria, outcomes, analyses) to relevant parties (eg, investigators, REC/IRBs, trial participants, trial registries, journals, regulators)	NA
Consent or assent	26a	Who will obtain informed consent or assent from potential trial participants or authorised surrogates, and how (see Item 32)	7
	26b	Additional consent provisions for collection and use of participant data and biological specimens in ancillary studies, if applicable	NA
Confidentiality	27	How personal information about potential and enrolled participants will be collected, shared, and maintained in order to protect confidentiality before, during, and after the trial	17

For peer review only - http://bmjopen.bmj.com/site/about/guidelines.xhtml

Declaration of interests	28	Financial and other competing interests for principal investigators for the overall trial and each study site	17
Access to data	29	Statement of who will have access to the final trial dataset, and disclosure of contractual agreements that limit such access for investigators	17
Ancillary and post-trial care	30	Provisions, if any, for ancillary and post-trial care, and for compensation to those who suffer harm from trial participation	NA
Dissemination policy	31a	Plans for investigators and sponsor to communicate trial results to participants, healthcare professionals, the public, and other relevant groups (eg, via publication, reporting in results databases, or other data sharing arrangements), including any publication restrictions	17
	31b	Authorship eligibility guidelines and any intended use of professional writers	NA
	31c	Plans, if any, for granting public access to the full protocol, participant-level dataset, and statistical code	NA
**Appendices**			
Informed consent materials	32	Model consent form and other related documentation given to participants and authorised surrogates	Appendix B, page 6
Biological specimens	33	Plans for collection, laboratory evaluation, and storage of biological specimens for genetic or molecular analysis in the current trial and for future use in ancillary studies, if applicable	NA

For peer review only - http://bmjopen.bmj.com/site/about/guidelines.xhtml

For peer review only

B. Tailored patient information leaflet

Name of patient : **Date:**
Agreed goal :
Date for next visit :

**Your blood pressure lowering drugs**			
Medicine name	When and how to take it?	What does it do?	Specific remarks

Reminders, habit-based strategies and/or involvement of family member

☐ Use pill boxes or a reminder app in your mobile phone.

☐ Make taking your blood pressure lowering drug(s) a part of your routine. Take it every day at around the same time. Choose a time that is easy to remember.

**Coping plan (first visit) ***	
IF it is (time of day):<br>……………………………………………………<br>And I am (where):<br>……………………………………………………<br>And I am (doing what):<br>……………………………………………………<br>THEN I will take (names of medication)<br>……………………………………………………<br>……………………………………………………	IF it is (time of day):<br>……………………………………………………<br>And I am (where):<br>……………………………………………………<br>And I am (doing what):<br>……………………………………………………<br>THEN I will take (names of medication)<br>……………………………………………………<br>……………………………………………………
**Follow-up after 1 month**	Succeeded ☐<br>If not, please explain why:<br>……………………………………………………

* Example: IF it is: first thing in the morning, and I am: in the kitchen, and I: am making my cup of tea, THEN: I will take my drug A

☐ Ask a family member to remind you to take your blood pressure lowering drug(s). I am going to ask: ……………………………………

☐ If you forgot to take the drug and it is less than 8 hours before your next dose, do not take a double dose of your blood pressure lowering drug(s) to make up for a forgotten dose. If it is more than 8 hours before your next dose, take the forgotten dose and take the next one as scheduled.

For peer review only - http://bmjopen.bmj.com/site/about/guidelines.xhtml

Knowledge about your blood pressure lowering drug(s)

- [ ] The purpose of your blood pressure lowering drug(s) is to reduce blood pressure level (short term effect) and to reduce the risk of developing heart disease in the future (long-term effect).
- [ ] Swallow your blood pressure lowering drug(s) with a drink of water.
- [ ] Common possible side effects of blood pressure lowering drug(s) are persistent dry cough, headache, dizziness, etc. If you get any side effects, please talk to your doctor or pharmacist. This includes any possible side effects not listed in this leaflet.

Motivation to take your blood pressure lowering drug(s)

- [ ] Your blood pressure lowering drug(s) only works when taken regularly as prescribed.
- [ ] Your blood pressure lowering drug(s) will not necessarily make you feel any different, but this does not mean it is not working.
- [ ] Do not stop taking your blood pressure lowering drug(s) without your doctor's consent unless you have an allergic reaction, e.g. rash or difficulty breathing. Abruptly stopping your blood pressure lowering drug(s) can cause a sudden increase in your blood pressure, which can be dangerous in some patients.
- [ ] Like all medicines, your blood pressure lowering drug(s) can cause side effects, but not everybody gets them. If you get any side effects, please talk to your doctor or pharmacist.
- [ ] Other:

____

____

____

Other drug-related problems

- [ ] If you have problem to refill your blood pressure lowering drug(s), please ask for help from your family or friend to accompany you to refill your medication.
- [ ] Other:

____

____

For peer review only - http://bmjopen.bmj.com/site/about/guidelines.xhtml

C. Counseling protocol in the intervention group

Baseline session

1. **Opening**

   *I would like to start with reviewing some of your medications and then we can talk about your experiences of taking them. (*Complete the "Your blood pressure lowering drugs' list at the top of the Tailored patient information leaflet*)*

2. **Identify any possible barriers using the flowchart at baseline and provide the intervention based on the adherence intervention wheel**

   Try to find out whether there are any barriers for not using the blood pressure lowering drug(s). Ask more if something is not clear. Once any barriers have been identified, deliver the intervention accordingly.

   1. **Forgetfulness: setting coping plans and reminder tools**

      Do not try to come directly with solutions and answers. Let the patient formulate his/her plans.

      1. *How often do you forget to take your blood pressure lowering drug(s)?*
      2. *Do you know when or why it happens that you forget to take your blood pressure lowering drug(s)?*

         (Possible answers: being busy, not able to make taking the drug a part of their routine, difficult to remember when to take the drug, forgot to take the drug when traveling, or lack of family support. Patients may have other answers regarding the reason for forgetfulness, please provide and adapt the solution accordingly.)
      3. *What time do you usually take each of your blood pressure lowering drug(s)?*
      4. *What would you like to see different about your current situation?*

For peer review only

5. *I would start with a simple way for reminding you to take your medicines such as set an alarm on your mobile phone or set a coping plan.* (Give the tailored leaflet and ask patients to write down their plans in the box Reminders).
6. *Could you ask your family to remind you to take your blood pressure lowering drug(s) regularly (if so, ask patient to write down name of family member in the box Involvement of family member)?*
7. Explain what to do when a dose is forgotten, for example*: If you forget to take a dose of your blood pressure lowering drug, take it as soon as you remember. However, if it less than 8 hours before your next dose is scheduled, skip the missed dose(s) and go back to your regular dosing schedule. Do not take a double dose of your blood pressure lowering drug to make up for a forgotten dose.*

2. Lack of knowledge: counseling and education

1. *Do you understand why you have to take your blood pressure lowering drug(s)?*
   a. If yes: *Can you explain this to me?*
   b. If no or insufficient: explain the purpose of blood pressure lowering drug(s) regarding short (blood pressure) and long-term effects (prevention of heart attack and stroke); when relevant also explain which side effects can occur and how to deal with them when they occur. For example:
      i. Short term effect: *The purpose of your blood pressure lowering drug(s) is to reduce your blood pressure by relaxing or widening*

For peer review only - http://bmjopen.bmj.com/site/about/guidelines.xhtml

your blood vessels, making your heartbeat more slowly, or flushing excess water and salt from the body through your pee.

ii. Long term effect*: The purpose of your blood pressure lowering drug(s) is to reduce the risk of developing heart disease in the future, such as heart attack and stroke.*

2. *Do you understand how you have to take your blood pressure lowering drug(s)?*
   a. If yes: *Can you explain this to me?*
   b. If no or insufficient: Explain when to take and how much to take.
3. *Could you explain what we have talked about so far?*
4. *Do you have more questions?*

3. Lack of motivation: counseling

1. *Do your blood pressure lowering drug(s) bother you in any way?*

   If yes: Explore in what way the drug bother the patient. If it is related to the high concerns and low necessity, continue with the following questions.

2. *Do you think that it is important to use blood pressure lowering drug(s)?*
   a. If yes: *Can you explain this to me?*
   b. If no or insufficient: Explain that the blood pressure lowering drug(s) only works when taken as prescribed. If patients answer that they did not feel anything changed after taking the drug, explain that blood pressure lowering drug(s) will not necessarily make them feel any different, but this does not mean the drug is not working. When relevant, also explain

For peer review only - http://bmjopen.bmj.com/site/about/guidelines.xhtml

the importance of the blood pressure lowering drug(s) in reducing blood pressure level and prevent future cardiovascular diseases.

3. *Do you have any concerns when using blood pressure lowering drug(s)?*
   a. If no: Continue with the following question.
   b. If yes: Explore what the concerns are. If it is related to side effects, try to reduce it by explaining the common possible side effects which is not dangerous and by convincing patients that not everybody experienced the side effects. For example, start with this statement:
   Most people will be able to take their medicines without any problems. However, a few people can have side effects from their medicines. Everyone is different, and there is no way to tell whether you will have a side effect from your medicine. Most people who have side effects will feel them soon after they start to take a new medicine, or a higher dose of their medicine. (Of note, most of the more serious side effects of blood pressure lowering drug(s) are rare, for example, severe allergic reactions may affect up to 1 in 1,000 people or severe skin disorders -a sudden, unexpected rash or burning- may affect up to 1 in 10,000 people).

   Continue with this statement to reduce concerns about becoming too dependent on blood pressure lowering drug(s):
   High blood pressure is a long-term problem that requires long-term treatment to keep it under control. For this reason, most people need to take tablets to keep their blood pressure down for the rest of their lives. However, a few people are able to bring their blood pressure down by

For peer review only - http://bmjopen.bmj.com/site/about/guidelines.xhtml

really sticking to changes in their lifestyle, take the drug as prescribed, and later may be able to reduce the dose or some of their tablets.

4. *Are you worried about any side effects in the long term?*
   a. If no: Continue with the following question.
   b. If yes: Explain that some side effects may disappear over time, or patients may get used to them. Most side effects of blood pressure lowering drug(s) are not dangerous. Patients can always talk to doctor or pharmacist. For example:
   If you start to feel different after taking your blood pressure lowering drug(s) in the long-term, please talk to your doctor or to me. Your doctor may be able to change your dose, or try a different medicine.

4. Other problems: explore and offer possible solutions

1. *Do you have any side effects that you think may be caused by your blood pressure lowering drug(s)?*
   If yes: please call the Puskesmas or come over to Puskesmas.
2. *Do you have any other problem with taking or collecting your blood pressure lowering drug(s)?*
   a. If yes: Possible answers are due to experiencing side effect, polypharmacy, financial issue, lack of access to CHC, or the use of traditional medicine. Patients may have other answers that are not listed here. Try to offer solutions according to their answer, for example, if patients have a difficulty to refill the drug in time, suggest them to ask for help from family of friend to accompany them to refill the blood pressure lowering drug(s).

For peer review only - http://bmjopen.bmj.com/site/about/guidelines.xhtml

b. If no: *Can you explain why you sometimes/often/always do not take your blood pressure lowering drug(s)?* (Some patients take less medication or lower dosages as 'self-management'; such patients may have good knowledge and motivation*)*

3. Closing

At the end of each counseling, involve patients in a goal setting process, for example to reduce forgetfulness. Ask their current blood pressure level and take it into account when discussing the goal. If patients already have a controlled blood pressure, explain that they can receive a reduced doses or reduced number of blood pressure lowering drug(s) (when relevant) in the future if they can maintain the blood pressure level under control. This must be done after discussing it first with the responsible doctor. After that, write the agreed goal at the top of the tailored leaflet for patients to take home. Please remind patients to take his/her leaflet in the next visit, and **make a copy of the leaflet for your own and the researchers to use**.

Follow-up session

1. Review and discuss patient's implementation and experiences with the discussed plans and recommendations.

1. For those who already adherent in the follow-up session: *You should be proud of all effort you have put into taking your blood pressure lowering drug(s) as prescribed to you.*
2. *What is your experience so far? How is it going?*

2. Identify all further barriers that were not yet addressed during the first session using the flowchart at 1-month follow up visit and provide the intervention based on the adherence intervention wheel.

For peer review only - http://bmjopen.bmj.com/site/about/guidelines.xhtml

Try to find out whether there are still any barriers for not using the blood pressure lowering drug(s). Ask more if something is not clear. Once any barriers have been identified, deliver the intervention accordingly (see baseline session).

For peer review only

For peer review only - http://bmjopen.bmj.com/site/about/guidelines.xhtml

APPENDIX

A. SPIRIT checklist

SPIRIT

STANDARD PROTOCOL ITEMS: RECOMMENDATIONS FOR INTERVENTIONAL TRIALS

For peer review only

Section/item	Item No	Description	Reported on page
**Administrative information**			
Title	1	Descriptive title identifying the study design, population, interventions, and, if applicable, trial acronym	1
Trial registration	2a	Trial identifier and registry name. If not yet registered, name of intended registry	2
	2b	All items from the World Health Organization Trial Registration Data Set	4-20
Protocol version	3	Date and version identifier	NA
Funding	4	Sources and types of financial, material, and other support	17
Roles and responsibilities	5a	Names, affiliations, and roles of protocol contributors	1, 17
	5b	Name and contact information for the trial sponsor	NA
	5c	Role of study sponsor and funders, if any, in study design; collection, management, analysis, and interpretation of data; writing of the report; and the decision to submit the report for publication, including whether they will have ultimate authority over any of these activities	17
	5d	Composition, roles, and responsibilities of the coordinating centre, steering committee, endpoint adjudication committee, data management team, and other individuals or groups overseeing the trial, if applicable (see Item 21a for data monitoring committee)	NA

For peer review only - http://bmjopen.bmj.com/site/about/guidelines.xhtml

**Introduction**			
Background and rationale	6a	Description of research question and justification for undertaking the trial, including summary of relevant studies (published and unpublished) examining benefits and harms for each intervention	4-5
	6b	Explanation for choice of comparators	10
Objectives	7	Specific objectives or hypotheses	5
Trial design	8	Description of trial design including type of trial (eg, parallel group, crossover, factorial, single group), allocation ratio, and framework (eg, superiority, equivalence, non-inferiority, exploratory)	6,7
**Methods: Participants, interventions, and outcomes**			
Study setting	9	Description of study settings (eg, community clinic, academic hospital) and list of countries where data will be collected. Reference to where list of study sites can be obtained	6
Eligibility criteria	10	Inclusion and exclusion criteria for participants. If applicable, eligibility criteria for study centres and individuals who will perform the interventions (eg, surgeons, psychotherapists)	6,7
Interventions	11a	Interventions for each group with sufficient detail to allow replication, including how and when they will be administered	7-10
	11b	Criteria for discontinuing or modifying allocated interventions for a given trial participant (eg, drug dose change in response to harms, participant request, or improving/worsening disease)	NA
	11c	Strategies to improve adherence to intervention protocols, and any procedures for monitoring adherence (eg, drug tablet return, laboratory tests)	14
	11d	Relevant concomitant care and interventions that are permitted or prohibited during the trial	7
Outcomes	12	Primary, secondary, and other outcomes, including the specific measurement variable (eg, systolic blood pressure), analysis metric (eg, change from baseline, final value, time to event), method of aggregation (eg, median, proportion), and time point for each outcome. Explanation of the clinical relevance of chosen efficacy and harm outcomes is strongly recommended	11-13
Participant timeline	13	Time schedule of enrolment, interventions (including any run-ins and washouts), assessments, and visits for participants. A schematic diagram is highly recommended (see Figure)	Figure 1

For peer review only - http://bmjopen.bmj.com/site/about/guidelines.xhtml

Sample size	14	Estimated number of participants needed to achieve study objectives and how it was determined, including clinical and statistical assumptions supporting any sample size calculations	14
Recruitment	15	Strategies for achieving adequate participant enrolment to reach target sample size	14
**Methods: Assignment of interventions (for controlled trials)**			
Allocation:			
Sequence generation	16a	Method of generating the allocation sequence (eg, computer-generated random numbers), and list of any factors for stratification. To reduce predictability of a random sequence, details of any planned restriction (eg, blocking) should be provided in a separate document that is unavailable to those who enrol participants or assign interventions	7
Allocation concealment mechanism	16b	Mechanism of implementing the allocation sequence (eg, central telephone; sequentially numbered, opaque, sealed envelopes), describing any steps to conceal the sequence until interventions are assigned	7
Implementation	16c	Who will generate the allocation sequence, who will enrol participants, and who will assign participants to interventions	7
Blinding (masking)	17a	Who will be blinded after assignment to interventions (eg, trial participants, care providers, outcome assessors, data analysts), and how	7
	17b	If blinded, circumstances under which unblinding is permissible, and procedure for revealing a participant's allocated intervention during the trial	NA
**Methods: Data collection, management, and analysis**			
Data collection methods	18a	Plans for assessment and collection of outcome, baseline, and other trial data, including any related processes to promote data quality (eg, duplicate measurements, training of assessors) and a description of study instruments (eg, questionnaires, laboratory tests) along with their reliability and validity, if known. Reference to where data collection forms can be found, if not in the protocol	11-14
	18b	Plans to promote participant retention and complete follow-up, including list of any outcome data to be collected for participants who discontinue or deviate from intervention protocols	12, 14
Data management	19	Plans for data entry, coding, security, and storage, including any related processes to promote data quality (eg, double data entry; range checks for data values). Reference to where details of data management procedures can be found, if not in the protocol	15

For peer review only - http://bmjopen.bmj.com/site/about/guidelines.xhtml

Statistical methods	20a	Statistical methods for analysing primary and secondary outcomes. Reference to where other details of the statistical analysis plan can be found, if not in the protocol	14
	20b	Methods for any additional analyses (eg, subgroup and adjusted analyses)	NA
	20c	Definition of analysis population relating to protocol non-adherence (eg, as randomised analysis), and any statistical methods to handle missing data (eg, multiple imputation)	14
**Methods: Monitoring**			
Data monitoring	21a	Composition of data monitoring committee (DMC); summary of its role and reporting structure; statement of whether it is independent from the sponsor and competing interests; and reference to where further details about its charter can be found, if not in the protocol. Alternatively, an explanation of why a DMC is not needed	NA
	21b	Description of any interim analyses and stopping guidelines, including who will have access to these interim results and make the final decision to terminate the trial	NA
Harms	22	Plans for collecting, assessing, reporting, and managing solicited and spontaneously reported adverse events and other unintended effects of trial interventions or trial conduct	15
Auditing	23	Frequency and procedures for auditing trial conduct, if any, and whether the process will be independent from investigators and the sponsor	NA
**Ethics and dissemination**			
Research ethics approval	24	Plans for seeking research ethics committee/institutional review board (REC/IRB) approval	17
Protocol amendments	25	Plans for communicating important protocol modifications (eg, changes to eligibility criteria, outcomes, analyses) to relevant parties (eg, investigators, REC/IRBs, trial participants, trial registries, journals, regulators)	NA
Consent or assent	26a	Who will obtain informed consent or assent from potential trial participants or authorised surrogates, and how (see Item 32)	7
	26b	Additional consent provisions for collection and use of participant data and biological specimens in ancillary studies, if applicable	NA
Confidentiality	27	How personal information about potential and enrolled participants will be collected, shared, and maintained in order to protect confidentiality before, during, and after the trial	17

For peer review only - http://bmjopen.bmj.com/site/about/guidelines.xhtml

Declaration of interests	28	Financial and other competing interests for principal investigators for the overall trial and each study site	17
Access to data	29	Statement of who will have access to the final trial dataset, and disclosure of contractual agreements that limit such access for investigators	17
Ancillary and post-trial care	30	Provisions, if any, for ancillary and post-trial care, and for compensation to those who suffer harm from trial participation	NA
Dissemination policy	31a	Plans for investigators and sponsor to communicate trial results to participants, healthcare professionals, the public, and other relevant groups (eg, via publication, reporting in results databases, or other data sharing arrangements), including any publication restrictions	17
	31b	Authorship eligibility guidelines and any intended use of professional writers	NA
	31c	Plans, if any, for granting public access to the full protocol, participant-level dataset, and statistical code	NA
**Appendices**			
Informed consent materials	32	Model consent form and other related documentation given to participants and authorised surrogates	Appendix B, page 6
Biological specimens	33	Plans for collection, laboratory evaluation, and storage of biological specimens for genetic or molecular analysis in the current trial and for future use in ancillary studies, if applicable	NA

For peer review only

For peer review only - http://bmjopen.bmj.com/site/about/guidelines.xhtml

For peer review only

BMJ Open

Targeted and tailored pharmacist-led intervention to improve adherence to antihypertensive drugs among patients with type 2 diabetes in Indonesia: study protocol of a cluster randomized controlled trial

Journal:	*BMJ Open*
Manuscript ID	bmjopen-2019-034507.R1
Article Type:	Protocol
Date Submitted by the Author:	25-Nov-2019
Complete List of Authors:	Alfian, Sofa; University of Groningen; Universitas Padjadjaran,<br>Abdulah, Rizky; Universitas Padjadjaran; Universitas Padjadjaran, Center of Excellence in Higher Education for Pharmaceutical Care Innovation<br>Denig, Petra; University Medical Centre Groningen; Medication Adherence Expertise Center of the northern Netherlands (MAECON)<br>van Boven, Job ; University Medical Centre Groningen; Medication Adherence Expertise Center of the northern Netherlands (MAECON)<br>Hak, Eelko; University of Groningen; Medication Adherence Expertise Center of the northern Netherlands (MAECON)
**Primary Subject Heading**:	Diabetes and endocrinology
Secondary Subject Heading:	Cardiovascular medicine
Keywords:	Diabetes & endocrinology < INTERNAL MEDICINE, medication adherence, Hypertension < CARDIOLOGY

SCHOLARONE™
Manuscripts

Targeted and tailored pharmacist-led intervention to improve adherence to antihypertensive drugs among patients with type 2 diabetes in Indonesia: study protocol of a cluster randomized controlled trial

Sofa D. Alfian[1,2,3*], Rizky Abdulah[2,3], Petra Denig[4,5], Job F. M. van Boven[4,5], Eelko Hak[1,5]

[1] University of Groningen, Groningen Research Institute of Pharmacy, Unit Pharmaco-Therapy, -Epidemiology & -Economics, Groningen, the Netherlands

[2] Universitas Padjadjaran, Faculty of Pharmacy, Department of Pharmacology and Clinical Pharmacy, Jatinangor, Indonesia

[3] Universitas Padjadjaran, Center of Excellence in Higher Education for Pharmaceutical Care Innovation, Jatinangor, Indonesia

[4] University of Groningen, University Medical Centre Groningen, Department of Clinical Pharmacy and Pharmacology, Groningen, the Netherlands

[5] Medication Adherence Expertise Center of the northern Netherlands (MAECON), Groningen, the Netherlands

*Corresponding author: Email address: s.d.alfian@rug.nl/sofa.alfian@unpad.ac.id

For peer review only

ABSTRACT

Introduction: Current intervention programs to improve drug adherence are either too complex or expensive for implementation and scale-up in low-middle income countries. The aim of this study is to assess the process and effects of implementing a low-cost, targeted and tailored pharmacist intervention among patients with type 2 diabetes who are non-adherent to antihypertensive drugs in a real-world primary care Indonesian setting.

Methods and analysis: A cluster randomized controlled trial with 3-month follow-up will be conducted in 10 community health centers (CHCs) in Indonesia. Type 2 diabetes patients aged 18 years and older who reported non-adherence to antihypertensive drugs according to the Medication Adherence Report Scale (MARS) are eligible to participate. Patients in CHCs randomized to the intervention group will receive a tailored intervention based on their personal adherence barriers. Interventions may include reminders, habit-based strategies, family support, counseling to educate and motivate patients, and strategies to address other drug-related problems. Interventions will be provided at baseline and at 1-month follow-up. Simple question-based flow-charts and an innovative adherence intervention wheel are provided to support the pharmacy staff. Patients in CHCs randomized to the control group will receive usual care based on the Indonesian guideline. Primary outcome is the between-group difference in medication adherence change from baseline to 3-month follow-up assessed by MARS. Secondary outcomes include changes in patients' blood pressure, their medication beliefs assessed by the Beliefs about Medicines Questionnaire (BMQ)-specific, as well as process characteristics of the intervention program from a pharmacist and patient perspective.

Ethics and dissemination: Ethical approval was obtained from the Ethical Committee of Universitas Padjadjaran, Indonesia and all patients will provide written informed consent prior to participation. The findings of the study will be disseminated through international conferences, one or more peer-reviewed journals, and reports to key stakeholders.

Trial registration: clinicaltrials.gov identifier: NCT04023734

For peer review only - http://bmjopen.bmj.com/site/about/guidelines.xhtml

Article Summary

Strengths and limitations of this study

- The pharmacist-led intervention program uses principles of targeting by screening for non-adherence and tailoring to the patients' personal adherence problems to enhance its potential effect. Simple question-based flow-charts and an innovative adherence intervention wheel are provided to support the pharmacy staff.
- The intervention aligns with the current workflow and resources in daily clinical practice of a lower-middle income country and will not require a substantial change to the current care system.
- This study is designed as a cluster randomized controlled trial to reduce risk of bias and contamination across study groups.
- The implementation process will be evaluated using the RE-AIM framework, which will enable us to carefully interpret the results and guide us when scaling up the intervention to include a larger population in Indonesia and other lower-middle income settings.
- As is common with many behavioral intervention studies, it is not possible to blind the researchers and pharmacists to the group allocation of patients.

For peer review only

For peer review only - http://bmjopen.bmj.com/site/about/guidelines.xhtml

INTRODUCTION

In patients with type 2 diabetes, pharmacological treatment of comorbid hypertension can substantially reduce the risk of cardiovascular complications.[1] However, while effective pharmacological treatment is available, adherence to antihypertensive medications in patients with type 2 diabetes is known to be suboptimal.[2] Notably, non-adherence to antihypertensive medications is associated with poor health outcomes and increased healthcare costs.[3] Therefore, effective intervention strategies to enhance adherence are urgently required.

Previous studies showed that patients may not take their medication for various reasons. Non-adherence could arise following a conscious decision after balancing the pros and cons of medication (intentional non-adherence), could be due to a lack of understanding the medication regimen, or due to forgetfulness (unintentional non-adherence).[4–7] The reasons underlying intentional and unintentional non-adherence are not entirely independent, and are heterogeneous. These reasons include lack of attention, lack of knowledge, high concerns and/or low necessity beliefs, which can reduce motivation.[4–7] In addition, there may be other drug-related problems, such as difficulties with intake or high costs, that can lead to non-adherence.[8,9] As such, there are no one-size-fits-all solutions to address non-adherence.

In developed countries with well-established healthcare systems, a wide variety of interventions to improve medication adherence have been developed.[10,11] Six main types of interventions can be identified: patient education, medication regimen management, clinical pharmacist consultation, cognitive behavioral therapies, medication-taking reminders, and incentives to promote adherence.[11] However, a Cochrane review showed that most interventions are often too complex and not particularly effective.[10] Additionally, in lower-middle income countries,

For peer review only - http://bmjopen.bmj.com/site/about/guidelines.xhtml

the paucity of healthcare and economic resources poses challenges to proper implementation of adherence enhancing interventions.

Non-adherence may be more efficiently improved if only patients who need it are targeted and interventions are tailored to patients' individual adherence barriers.[12,13] Previous studies further suggested that effective interventions to improve adherence were led by a pharmacist, delivered face-to-face, administered directly to patients, and behaviourally-targeted compared to cognitively-targeted interventions.[14] In Southeast Asia, however, pharmacy services can be hampered by inadequate training of pharmacy staff.[15] Furthermore, adherence interventions may not be sustained over time due to a lack of resources to maintain them. Thus, to improve adherence to antihypertensive drugs in patients with type 2 diabetes, a low-cost, targeted and tailored pharmacist-led intervention that can be integrated into the community pharmacy workflow is required.

The objectives of this study are (1) to assess the effect of a targeted and tailored pharmacist-led intervention on medication adherence to antihypertensive drugs among type 2 diabetes patients, (2) to assess the effect of the intervention on blood pressure level and medication beliefs, (3) to evaluate the implementation of the intervention from a pharmacist and patient perspective, and (4) to assess the effects of the intervention across different subgroups of patients.

For peer review only - http://bmjopen.bmj.com/site/about/guidelines.xhtml

METHODS AND ANALYSIS

This protocol was developed in accordance with the CONSORT 2010 statement for cluster randomized trials[16] and reported according to the SPIRIT checklist[17] (Supplementary appendix A). We will use the RE-AIM framework[18] to evaluate the implementation process of the intervention.

Study design and setting

This study is a 3-month cluster randomized controlled trial with two parallel arms and will be performed in Bandung City, Indonesia from August to December 2019 (Figure 1). Clusters of randomization are community health centres (CHCs), locally called *Puskesmas*. *Puskemas* are primary health care centres at the sub-district level, with each centre staffed with medical doctors, nurses, midwives, and pharmacists.

Participants

A total of 10 CHCs will be purposively selected based on a sufficient number of type 2 diabetes patients with hypertension. The principle investigator (PI) will introduce and explain the study to the pharmacists and physicians in the CHCs. In each CHC, one pharmacist will be included. In Indonesia, one of the pharmacist's responsibilities during routine clinical practice is to counsel patients with chronic diseases on their medication use, often performed in a counselling room which is separated from the drug counter.[19] Screening for patients' eligibility will be conducted by the pharmacist during regular outpatient visits. Once a patient is deemed eligible, the pharmacist will inform the PI or research assistant to approach the patient and briefly explain the study, and ask to sign informed consent (Supplementary appendix B). Patients will be eligible if they meet the following inclusion criteria: (i) at least 18 years old, (ii) diagnosed with

For peer review only - http://bmjopen.bmj.com/site/about/guidelines.xhtml

type 2 diabetes for at least one year based on patient's medical record, (iii) using at least one antihypertensive drug in the last three months, (iv) have signed informed consent, and (v) have suboptimal medication adherence to antihypertensive drugs according to the Medication Adherence Report Scale (MARS score <20; MARS scores range from 5 to 25). Of note, our previous work in four regions in Indonesia (Bandung City, Yogyakarta City, Makassar City, and Samarinda City) showed that half of patients were non-adherent, with a MARS score < 20. Patients with severe mental or physical constraints, pregnancy or in the lactation period, illiterate in the Indonesian language, enrolment in another intervention study, and those not responsible for taking their own medication will be excluded.

Randomization and blinding

We will use cluster randomization at the CHCs level to reduce risk of bias and contamination across study groups.[20] The PI will randomize the CHCs into the intervention or control group in a 1:1 ratio. The randomization sequence will be generated using a random number generator. Given the nature of the study design, both pharmacists and the PI cannot be blinded to the group assignment.

Intervention

Patients in the five CHCs randomized to the intervention group who were screened as non-adherent to their antihypertensive drugs will receive a tailored pharmacist-led intervention during two sessions (at baseline and at 1-month follow-up) in addition to usual care. Both will be regular outpatient visits, when patients collect their medication. The intervention will be low-cost, align with the current CHC workflow and will not require a substantial change to the

For peer review only - http://bmjopen.bmj.com/site/about/guidelines.xhtml

For peer review only

current system. Before the study started, the intervention steps and materials were piloted in two pharmacies and optimized in an iterative process.

Intervention at baseline (first session)

Before dispensing antihypertensive drugs during the first session, the pharmacist will discuss patient-specific barrier(s) for medication adherence based on their responses to the MARS questionnaire and three additional questions, which are derived from the Brief Medication Questionnaire[21] (Figure 2). The intervention strategy will then be tailored to the identified adherence problems. Based on current literature[22–24], we defined four non-adherence problems that can be addressed by the community pharmacists, i.e. (1) forgetfulness, (2) lack of knowledge, (3) lack of motivation and/or (4) other drug-related problems. Of note, patients might need a combined intervention strategy to address all experienced problems. The four non-adherence problems and recommended intervention strategies are specified in Table 1. The session will end with involving patients in goal setting and writing the agreed goal at the top of the personalized leaflet. Pharmacists will remind patients to take his/her leaflet to the next visit.

Table 1 Non-adherence problems and recommended intervention strategies.

Non-adherence problems	Intervention strategies
1. Forgetfulness	Strategies to cope with forgetfulness include reminders, habit-based strategies and/or involvement of family members, and improving knowledge on what to do when a dose is forgotten.[23] The use of a reminder tool or pill boxes will be encouraged and a reminder app can be implemented if the patient owns a mobile phone. The habit-based strategy will be delivered through a personalized leaflet, which is tailored to patient's daily routine (Supplementary appendix C). Patients will be asked

For peer review only - http://bmjopen.bmj.com/site/about/guidelines.xhtml

	to identify the appropriate place and time to take their medication, and an activity they conduct every day that could serve as a prompt or cue to take their medication.[25] Patients will be asked to write coping plans to formulate their own "if–then" plans for the daily doses of their antihypertensive drug(s).[25] Moreover, patients will be asked to choose a family member to become their treatment supporter and to write down the name of family member on the personalized leaflet. This individual will be asked to support the patient to take the antihypertensive drugs. Pharmacists will keep a copy of the leaflet and remind patients to take his/her personalized leaflet to the next visit.
2. Lack of knowledge	Patient counseling by the pharmacist to cope with lack of knowledge may focus on educating the patient about the purpose of the medication, when and how to take the medication, the need for long-term use, the importance of medication adherence, and how to deal with possible side effects. To explore which education is needed, the patient will be asked whether they know why and how to take their medication. The teach-back method will be used, where the patient is asked to explain the pharmacist what he/she has understood after receiving the education.[26]
3. Lack of motivation	Counseling to cope with lack of motivation will focus on exploring and discussing the patients' concerns and necessity beliefs. This method is called motivational interviewing.[27] This is done by asking a first question about whether the medication bothers the patient. Follow-up on this question can focus on reducing any concerns or low necessity beliefs (e.g., when patients are bothered by the medication because they think the medication is not needed or are afraid of side-effects).
4. Other drug-related problems	Counseling to address other drug-related problems will focus on exploring other problems underlying non-adherence, for example, experiencing side effects, costs, polypharmacy, difficulty to refill antihypertensive drugs in time, or medication intake problems, and offering solutions/alternatives when possible.

For peer review only

Interventions at follow-up (second session)

The follow-up session will be conducted one month after the baseline session, when patients refill their medication at the next regular outpatient visit (Figure 3). The purpose of the follow-up session is (1) to evaluate the short-term effect of the intervention and discuss the patients' implementation of and experiences with the offered information and recommendations, and (2) to address non-adherence problems that were not yet addressed during the first session. Where needed, the pharmacist, together with patient, can make changes to the coping plan and discuss additional interventions. The session will end with involving patients in goal setting and writing the agreed goal at the top of the personalized leaflet.

Pharmacist training

As the quality of the intervention will depend on the competences and skills of the pharmacist, treatment integrity will be enhanced by an obligatory communication training focusing on how to elicit and classify barriers to adherence, the teach-back method and motivational interviewing, and by providing supportive material as part of the intervention (Figure 4).

Pharmacists and patients participating in this study will be compensated with a modest souvenir at the end of the study for their time and effort.

Control group

Patients in the five CHCs randomized to the control group will receive pharmacist counseling based on the Indonesian guideline of pharmacy practice (PMK No.74/2016).[19] At each visit, they can receive information about the quantity and dose of the dispensed drugs, when and how to use and store the drugs, side effects and how to deal with them, the importance of medication adherence, and confirming if the patient understands how to take medications correctly. Patients

For peer review only - http://bmjopen.bmj.com/site/about/guidelines.xhtml

For peer review only

in the control group who were screened as non-adherent to their antihypertensive drugs by the research assistant at baseline will complete the assessments at the same time points as those in the intervention group.

Outcomes

Primary outcome

The primary outcome is the difference between the intervention and the control group in change in MARS score from baseline (T0) to 3-month follow-up (T2). The Indonesian version of the MARS showed to be valid (correlation value of each question to the total score > 0.396) and reliable (Cronbach α coefficient of 0.803).[28] Patients will indicate how often each statement applied to them in the last three months on a 5-point Likert scale ranging from always (score 1) to never (score 5). Items are summed to obtain a total score ranging from 5 to 25 (Horne R. The Medication Adherence Report Scale (MARS): a new measurement tool for eliciting patients' reports of non-adherence).

Secondary outcomes

1. Blood pressure level

   Within and between patient changes in blood pressure (BP) level (systolic blood pressure [SBP] and diastolic blood pressure [DBP]) will be assessed. BP measurements will be performed by a nurse who is blinded to the group assignment at baseline (T0), 1-month (T1), and 3-month follow-up (T2).

2. Medication beliefs

   Within patient changes on beliefs about medication will be assessed using the Beliefs about Medicines Questionnaire (BMQ)-specific at baseline (T0) and 3-month follow-up

For peer review only - http://bmjopen.bmj.com/site/about/guidelines.xhtml

For peer review only

(T2). The Indonesian version of the BMQ-specific showed to be valid (correlation value of each question to the total score > 0.530) and reliable (Cronbach α coefficient of 0.835 and 0.811 for necessity and concerns beliefs, respectively). The BMQ-specific contains five items about necessity beliefs (e.g. "My health at present depends on my blood pressure-lowering medicines"), five items about concern beliefs (e.g. "I sometimes worry about becoming too dependent on my blood pressure-lowering medicines"), and one item about side effects (e.g. 'My blood pressure-lowering medicines gives me unpleasant side effects). All items have a 5-point Likert scale ranging from strongly disagree to strongly agree with an overall range from 5 (low necessity, low concern) to 25 (high necessity, high concern). A necessity–concern differential score will be calculated by subtracting the scores of the concerns scale from the necessity scale (range −20 to 20). A positive differential score indicates stronger beliefs in the necessity, while a negative score indicates stronger concerns.[29] The additional item about side effects will be analyzed separately due to its known role in non-adherence.[22,30]

3. Process evaluation

We will conduct a process evaluation to assess other parts of the RE-AIM framework. In short, the RE-AIM framework has been developed to evaluate public health interventions assessing five dimensions (reach, efficacy, adoption, implementation and maintenance) at multiple levels (e.g., individual or organization).[18] *Reach* will be assessed by measuring the participation rates and representativeness of patients who participate in this study. In case a patient refuses or discontinues to participate in this study, the patient's age, gender, and BP lowering drugs the patient uses will be recorded by research assistants. This information is used to calculate the participation rate and assess differences between responders and non-responders. To determine representativeness, the patients'

For peer review only - http://bmjopen.bmj.com/site/about/guidelines.xhtml

demographics will be compared to census demographics in Bandung City, Indonesia. *Adoption* will be evaluated by assessing the proportion and representativeness of CHCs who participate in this study, and exploring pharmacists' and patients' satisfaction with and willingness to use various parts of the intervention. *Implementation* will be evaluated by determining whether the intervention was delivered as intended and exploring pharmacists' and patients' suggestions for future implementation. *Maintenance* will be assessed by determining whether the intervention can be maintained and the willingness of pharmacists and payers to continue the intervention as part of routine clinical practice. We will use focus group discussions at 1-month (T1) follow-up and an evaluation survey (based on a previously used survey[31]) at 3-month follow-up (T2) to explore pharmacists' and patients' adoption, implementation, and willingness to maintain the intervention, respectively.

Baseline participant and CHC characteristics

Participants' baseline characteristics, including sociodemographic and clinical-related factors, will be obtained. Sociodemographic factors are self-reported and include age at the completion of the questionnaire, gender, highest level of education completed (no formal education/elementary high school, junior high school, senior high school, or university), and type of health insurance. Type of health insurance will be classified as those whose insurance premium was paid by the government (BPJS-PBI), those whose insurance premium was paid by the patients themselves (BPJS-Non PBI), or those without health insurance. Clinical-related factors include time since diagnosis of diabetes and hypertension (years), diabetes complication(s) that developed after the diagnosis of diabetes, and types and number of concomitant medications. The following diabetes complications will be considered: cardiovascular conditions, cerebrovascular conditions, nephropathy, retinopathy, neuropathy,

For peer review only - http://bmjopen.bmj.com/site/about/guidelines.xhtml

and diabetic foot problems. Clinical-related factors will be collected by research assistants using a predefined data collection form. Furthermore, organizational information of each CHC (number of medical doctors, pharmacists, nurses, and average number of diabetes patients with and without hypertension visits per month) and pharmacist characteristics (age, gender, and working experience in community pharmacy [years]) will be collected by research assistants using a predefined data collection form.

Treatment fidelity

Treatment fidelity will be addressed by providing a checklist of items that pharmacists need to do at each patient visit and a counselling protocol for the intervention group (Supplementary appendix D). Pharmacists will be asked to complete the checklist after each visit with a study participant. The completed checklists will be collected on a weekly basis and used to calculate an overall fidelity score. Minor feedback suggestions from the PI to pharmacists will be made if needed.

Sample size calculation

The sample size calculation is based on the formula for cluster randomized trials, powered on the primary outcome.[32] We want to be able to detect a difference between the intervention and control group in change in adherence score of at least 2.5 points with an expected standard deviation of 3.8 points and assuming an intra-cluster correlation coefficient within CHCs of 0.014, as calculated based on previous work from our group (manuscript in preparation). A sample size of 41 non-adherent patients in each study arm (intervention and control group) would allow for 80% power to detect this difference using a two-sided test at the 5% level of significance. Assuming non-adherence rates of 50%[33] and a dropout rate of 20%, we will recruit at least 100 patients in each group, giving a total of 200 patients from 10 CHCs (20 per CHC)

For peer review only - http://bmjopen.bmj.com/site/about/guidelines.xhtml

For peer review only

that need to complete the MARS screening. Recruiting at least 20 patients per CHC is feasible since the average number of diabetes patients with hypertension visiting a CHC is around 30 patients per month.

Planned statistical analysis

Data analysis will be performed based on the intention-to-treat principle. Descriptive statistics will be used to summarize the baseline characteristics. To control for the effects of cluster randomization, group changes will be compared (individual CHCs will be treated as a random effect) using multivariate mixed linear and nonlinear regression for normally and non-normally distributed data, respectively. Point estimates estimated from cluster-adjusted models will be reported with 95% confidence intervals. We will conduct subgroup analysis using data stratification on diabetes complications and number of concomitant medications to assess the effect of the intervention. All tests will be two-tailed and $p<0.05$ will be considered statistically significant. All statistical analyses will be carried out using SPSS software (version 25.0; IBM, Armonk, NY, USA).

Data management

Data from the questionnaires and case report forms will be entered by the PI using a unique identifier that is provided for each participant into SPSS software version 25 and data forms will be stored on a password-protected computer.

Adverse event reporting

It is possible that a participant identifies a medication-related issue during pharmacist counseling, either in the intervention or control group. Although this is unlikely to be a result

For peer review only - http://bmjopen.bmj.com/site/about/guidelines.xhtml

from the study, the patient's doctor will be contacted if the pharmacist and/or researchers have concerns requiring immediate intervention.

Patient and public involvement

Patients and public were not involved in the development of the research question or outcome measures. Patients will be involved during the conduct of the study by giving feedback to tailor the intervention based on their personal adherence barrier(s). In addition, patients will be asked to complete an evaluation survey, including questions about the intervention. Patients will be given contact details of the PI to request the results of the study.

For peer review only

For peer review only - http://bmjopen.bmj.com/site/about/guidelines.xhtml

ETHICS AND DISSEMINATION

Ethical approval for this study was obtained from the Ethical Committee of Universitas Padjadjaran, Indonesia (No. 859/UN6.KEP/EC/2019). All results will be stored securely and will be available to authorized individuals for analysis and reporting purposes only. Data will be published in a form that does not identify patients in any way. To maintain patients' anonymity, a unique identifier will be used to match patients' data across baseline and follow-up. Patients are free to withdraw from the study at any time. The findings of the study will be disseminated through international conferences, one or more peer-reviewed journals, and reports to key stakeholders.

Authors' contributions

SDA wrote the first draft of this protocol. SDA, RA, JFMvB, PD, and EH participated in the design of the study and contributed to the revision of the study protocol. All authors approved the final manuscript.

Funding

SDA is supported by a scholarship from the Indonesia Endowment Fund for Education (LPDP No: PRJ-2361/LPDP/2015). This funding body did not have any role in designing the study, in writing this article, and in deciding to submit it for publication.

Competing interests

None declared.

For peer review only

For peer review only - http://bmjopen.bmj.com/site/about/guidelines.xhtml

REFERENCES

1. American Diabetes Association. 10. Cardiovascular Disease and Risk Management: *Standards of Medical Care in Diabetes—2019*. Diabetes Care. 2019;42(Supplement 1):S103-23.

2. Ledur PS, Leiria LF, Severo MD, et al. Perception of uncontrolled blood pressure and non-adherence to anti-hypertensive agents in diabetic hypertensive patients. J Am Soc Hypertens. 2013;7(6):477-83.

3. Khan R, Socha-Dietrich K. Investing in medication adherence improves health outcomes and health system efficiency: adherence to medicines for diabetes, hypertension, and hyperlipidaemia. OECD Heal Work Pap. 2018;(105).

4. Rees G, Leong O, Crowston JG, Lamoureux EL. Intentional and unintentional nonadherence to ocular hypotensive treatment in patients with glaucoma. Ophthalmology. 2010;117(5):903-8.

5. Iihara N, Kurosaki Y, Miyoshi C, Takabatake K, Morita S, Hori K. Comparison of individual perceptions of medication costs and benefits between intentional and unintentional medication non-adherence among Japanese patients. Patient Educ Couns. 2008;70(2):292-9.

6. Wroe AL. Intentional and unintentional nonadherence: a study of decision making. J Behav Med. 2002;25(4):355-72.

7. Clifford S, Barber N, Horne R. Understanding different beliefs held by adherers, unintentional nonadherers, and intentional nonadherers: Application of the Necessity-Concerns Framework. J Psychosom Res. 2008;64(1):41-6.

8. Sabate E. *Adherence to Long-Term Therapies: Evidence for Action*. World Health Organization; 2003.

9. Mathes T, Jaschinski T, Pieper D. Adherence influencing factors - a systematic review of systematic reviews. Arch Public Heal. 2014;72(1):1-9.

10. Nieuwlaat R, Wilczynski N, Navarro T, et al. Interventions for enhancing medication adherence. Cochrane Database Syst Rev. 2014;(11).

11. Kini V, Ho PM. Interventions to improve medication adherence. JAMA. 2018;320(23):2461.

12. Cutrona SL, Choudhry NK, Fischer MA, et al. Targeting cardiovascular medication adherence interventions. J Am Pharm Assoc. 2012;52(3):381-97.

13. van Boven JFM, Stuurman-Bieze AGG, Hiddink EG, Postma MJ, Vegter S. Medication Monitoring and Optimization: A Targeted Pharmacist Program for Effective and Cost-Effective Improvement of Chronic Therapy Adherence. J Manag Care Pharm. 2014;20(8):786-92.

14. Conn VS, Ruppar TM. Medication adherence outcomes of 771 intervention trials: Systematic review and meta-analysis. Prev Med (Baltim). 2017;99:269-76.

15. Hermansyah A, Sainsbury E, Krass I. Community pharmacy and emerging public

For peer review only - http://bmjopen.bmj.com/site/about/guidelines.xhtml

health initiatives in developing Southeast Asian countries: a systematic review. Health Soc Care Community. 2016;24(5):e11-22.

16. Campbell MK, Piaggio G, Elbourne DR, Altman DG. Consort 2010 statement: Extension to cluster randomised trials. BMJ. 2012;345(7881):1-21.

17. Chan A-W, Tetzlaff JM, Gøtzsche PC, et al. SPIRIT 2013 explanation and elaboration: guidance for protocols of clinical trials. BMJ. 2013;346:e7586.

18. Glasgow RE, Vogt TM, Boles SM. Evaluating the public health impact of health promotion interventions: the RE-AIM framework. Am J Public Health. 1999;89(9):1322-7.

19. Indonesia KKR. *Peraturan Menteri Kesehatan Republik Indonesia Nomor 74 Tahun 2016 Tentang Standar Pelayanan Kefarmasian Di Puskesmas*. Jakarta; 2016. http://jdih.pom.go.id/produk/peraturan menteri/PMK_No._74_ttg_Standar_Pelayanan_Kefarmasian_di_PUSKESMAS_.pdf. Accessed February 22, 2019.

20. Puffer S, Torgerson DJ, Watson J. Cluster randomized controlled trials. J Eval Clin Pract. 2005;11(5):479-83.

21. Svarstad BL, Chewning BA, Sleath BL, Claesson C. The Brief Medication Questionnaire: a tool for screening patient adherence and barriers to adherence. Patient Educ Couns. 1999;37(2):113-24.

22. van der Laan DM, Elders PJM, Boons CCLM, Beckeringh JJ, Nijpels G, Hugtenburg JG. Factors associated with antihypertensive medication non-adherence: a systematic review. J Hum Hypertens. 2017;31(11):687-94.

23. van der Laan DM, Elders PJM, Boons CCLM, Nijpels G, Hugtenburg JG. Factors associated with nonadherence to cardiovascular medications. J Cardiovasc Nurs. 2019;34(4):344-52.

24. van Boven JFM, Lavorini F, Dekhuijzen PNR, Blasi F, Price DB, Viegi G. Urging Europe to put non-adherence to inhaled respiratory medication higher on the policy agenda: a report from the First European Congress on Adherence to Therapy. Eur Respir J. 2017;49(5).

25. Meslot C, Gauchet A, Hagger MS, Chatzisarantis N, Lehmann A, Allenet B. A randomised controlled trial to test the effectiveness of planning strategies to improve medication adherence in patients with cardiovascular disease. Appl Psychol Heal Well-Being. 2017;9(1):106-29.

26. Dinh HTT, Bonner A, Clark R, Ramsbotham J, Hines S. The effectiveness of the teach-back method on adherence and self-management in health education for people with chronic disease: a systematic review. JBI Database Syst Rev Implement Reports. 2016;14(1):210.

27. Rollnick S, Miller WR, Arkowitz H, Westra HA, Butler CC. *Motivational Interviewing in Health Care: Helping Patients Change Behavior*. New York: The Guilford Press; 2008. http://www.monarchsystem.com/wp-content/uploads/2012/06/Motivational-Interviewing-in-Health-Care-Resource-Book.pdf.

For peer review only

For peer review only - http://bmjopen.bmj.com/site/about/guidelines.xhtml

28. Alfian R, Putra AMP. Uji validitas dan reliabilitas kuesioner medication adherence report scale (MARS) terhadap pasien diabetes mellitus [in Bahasa Indonesia]. J Ilm Ibnu Sina. 2017;2:176-83.

29. Horne R, Weinman J, Hankins M. The beliefs about medicines questionnaire: The development and evaluation of a new method for assessing the cognitive representation of medication. Psychol Heal. 1999;14(1):1-24.

30. Birtcher K. When compliance is an issue—how to enhance statin adherence and address adverse effects. Curr Atheroscler Rep. 2015;17(1). https://link.springer.com/content/pdf/10.1007%2Fs11883-014-0471-8.pdf. Accessed February 22, 2019.

31. Stuurman-Bieze AGG, Hiddink EG, van Boven JFM, Vegter S. Proactive Pharmaceutical Care Interventions Improve Patients' Adherence to Lipid-Lowering Medication. Ann Pharmacother. 2013;47(11):1448-56.

32. Rutterford C, Copas A, Eldridge S. Methods for sample size determination in cluster randomized trials. Int J Epidemiol. 2015;44(3):1051-67.

33. Adikusuma W, Perwitasari DA, Supadmi W. Evaluasi kepatuhan pasien diabetes melitus tipe 2 di Rumah Sakit Umum PKU Muhammadiyah Bantul, Yogyakarta [in Bahasa Indonesia]. Media Farm. 2014;11(2):208-20.

For peer review only

Figures

Figure 1 Consort flow chart

Abbreviation: BP: Blood pressure; CHC: Community health centre; MARS = Medication adherence report scale; BMQ-specific = Beliefs about medicines questionnaire-specific; FGD: Focus group discussions

Figure 2 Flow chart of a targeted and tailored intervention at baseline visit (T0)

Type of intervention: 1 = Reminders, habit-based strategies and/or involvement of family member; 2 = Counseling to increase knowledge (teach-back method); 3 = Counseling to increase motivation; 4 = Explore/address other drug related problems
Abbreviation: MARS: Medication adherence report scale

Figure 3 Flow chart of a targeted and tailored intervention at the one-month follow up visit (T1)

Type of intervention: 2 = Counseling to increase knowledge (teach-back method); 3 = Counseling to increase motivation; 4 = Explore/address other drug related problems
Abbreviation: MARS: Medication adherence report scale

Figure 4 Proposed adherence intervention wheel as supportive material for pharmacists in the intervention group

For peer review only

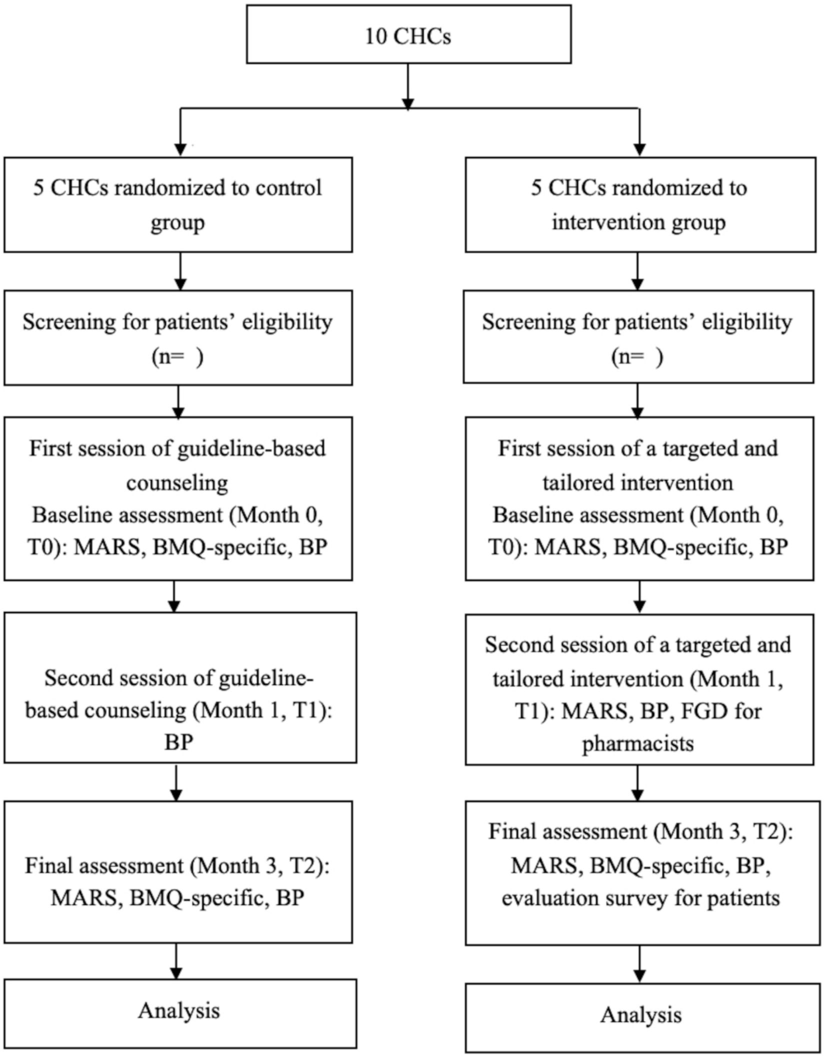

Figure 1 Consort flow chart
Abbreviation: BP: Blood pressure; CHC: Community health centre; MARS = Medication adherence report scale; BMQ-specific = Beliefs about medicines questionnaire-specific; FGD: Focus group discussions

236x270mm (300 x 300 DPI)

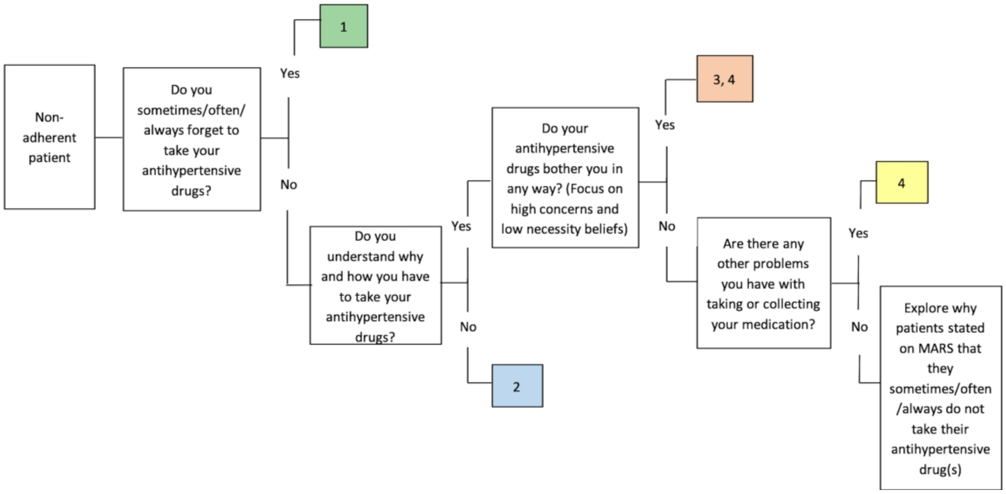

Figure 2 Flow chart of a targeted and tailored intervention at baseline visit (T0)
Type of intervention: 1 = Reminders, habit-based strategies and/or involvement of family member; 2 = Counseling to increase knowledge (teach-back method); 3 = Counseling to increase motivation; 4 = Explore/address other drug related problems
Abbreviation: MARS: Medication adherence report scale

289x140mm (300 x 300 DPI)

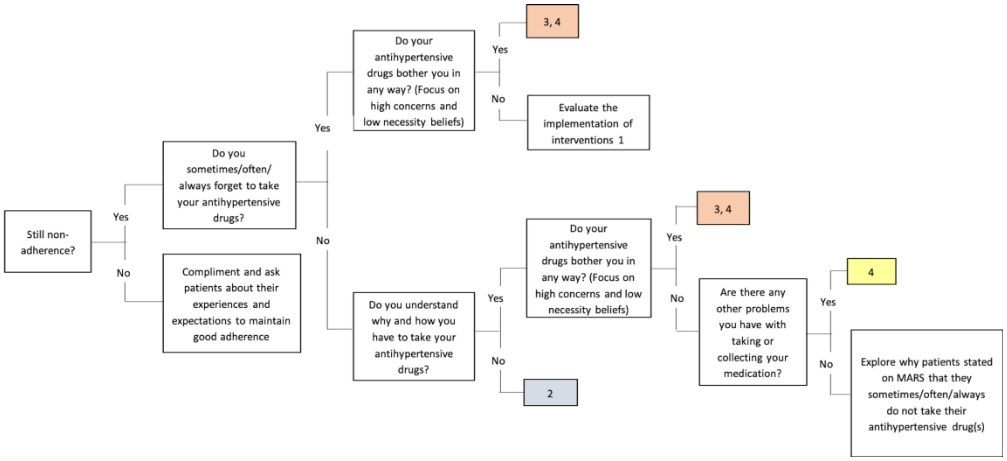

Figure 3 Flow chart of a targeted and tailored intervention at the one-month follow up visit (T1)
Type of intervention: 2 = Counseling to increase knowledge (teach-back method); 3 = Counseling to increase motivation; 4 = Explore/address other drug related problems
Abbreviation: MARS: Medication adherence report scale

307x139mm (300 x 300 DPI)

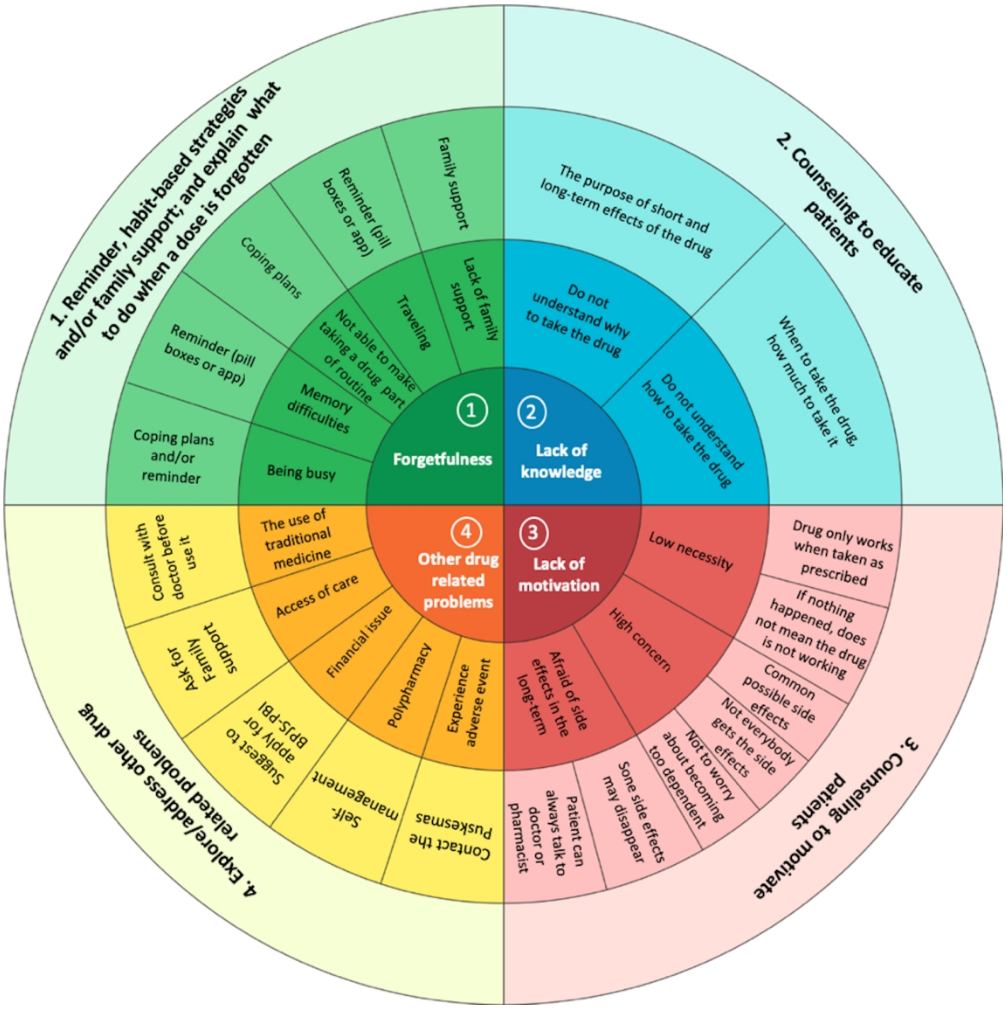

Figure 4 Proposed adherence intervention wheel as supportive material for pharmacists in the intervention group

159x159mm (300 x 300 DPI)

APPENDIX

A. SPIRIT checklist

SPIRIT
STANDARD PROTOCOL ITEMS: RECOMMENDATIONS FOR INTERVENTIONAL TRIALS

Section/item	Item No	Description	Reported on page
**Administrative information**			
Title	1	Descriptive title identifying the study design, population, interventions, and, if applicable, trial acronym	1
Trial registration	2a	Trial identifier and registry name. If not yet registered, name of intended registry	2
	2b	All items from the World Health Organization Trial Registration Data Set	4-20
Protocol version	3	Date and version identifier	NA
Funding	4	Sources and types of financial, material, and other support	17
Roles and responsibilities	5a	Names, affiliations, and roles of protocol contributors	1, 17
	5b	Name and contact information for the trial sponsor	NA
	5c	Role of study sponsor and funders, if any, in study design; collection, management, analysis, and interpretation of data; writing of the report; and the decision to submit the report for publication, including whether they will have ultimate authority over any of these activities	17
	5d	Composition, roles, and responsibilities of the coordinating centre, steering committee, endpoint adjudication committee, data management team, and other individuals or groups overseeing the trial, if applicable (see Item 21a for data monitoring committee)	NA

For peer review only - http://bmjopen.bmj.com/site/about/guidelines.xhtml

**Introduction**			
Background and rationale	6a	Description of research question and justification for undertaking the trial, including summary of relevant studies (published and unpublished) examining benefits and harms for each intervention	4-5
	6b	Explanation for choice of comparators	10
Objectives	7	Specific objectives or hypotheses	5
Trial design	8	Description of trial design including type of trial (eg, parallel group, crossover, factorial, single group), allocation ratio, and framework (eg, superiority, equivalence, non-inferiority, exploratory)	6,7
**Methods: Participants, interventions, and outcomes**			
Study setting	9	Description of study settings (eg, community clinic, academic hospital) and list of countries where data will be collected. Reference to where list of study sites can be obtained	6
Eligibility criteria	10	Inclusion and exclusion criteria for participants. If applicable, eligibility criteria for study centres and individuals who will perform the interventions (eg, surgeons, psychotherapists)	6,7
Interventions	11a	Interventions for each group with sufficient detail to allow replication, including how and when they will be administered	7-10
	11b	Criteria for discontinuing or modifying allocated interventions for a given trial participant (eg, drug dose change in response to harms, participant request, or improving/worsening disease)	NA
	11c	Strategies to improve adherence to intervention protocols, and any procedures for monitoring adherence (eg, drug tablet return, laboratory tests)	14
	11d	Relevant concomitant care and interventions that are permitted or prohibited during the trial	7
Outcomes	12	Primary, secondary, and other outcomes, including the specific measurement variable (eg, systolic blood pressure), analysis metric (eg, change from baseline, final value, time to event), method of aggregation (eg, median, proportion), and time point for each outcome. Explanation of the clinical relevance of chosen efficacy and harm outcomes is strongly recommended	11-13
Participant timeline	13	Time schedule of enrolment, interventions (including any run-ins and washouts), assessments, and visits for participants. A schematic diagram is highly recommended (see Figure)	Figure 1

For peer review only - http://bmjopen.bmj.com/site/about/guidelines.xhtml

Sample size	14	Estimated number of participants needed to achieve study objectives and how it was determined, including clinical and statistical assumptions supporting any sample size calculations	14
Recruitment	15	Strategies for achieving adequate participant enrolment to reach target sample size	14
**Methods: Assignment of interventions (for controlled trials)**			
Allocation:			
Sequence generation	16a	Method of generating the allocation sequence (eg, computer-generated random numbers), and list of any factors for stratification. To reduce predictability of a random sequence, details of any planned restriction (eg, blocking) should be provided in a separate document that is unavailable to those who enrol participants or assign interventions	7
Allocation concealment mechanism	16b	Mechanism of implementing the allocation sequence (eg, central telephone; sequentially numbered, opaque, sealed envelopes), describing any steps to conceal the sequence until interventions are assigned	7
Implementation	16c	Who will generate the allocation sequence, who will enrol participants, and who will assign participants to interventions	7
Blinding (masking)	17a	Who will be blinded after assignment to interventions (eg, trial participants, care providers, outcome assessors, data analysts), and how	7
	17b	If blinded, circumstances under which unblinding is permissible, and procedure for revealing a participant’s allocated intervention during the trial	NA
**Methods: Data collection, management, and analysis**			
Data collection methods	18a	Plans for assessment and collection of outcome, baseline, and other trial data, including any related processes to promote data quality (eg, duplicate measurements, training of assessors) and a description of study instruments (eg, questionnaires, laboratory tests) along with their reliability and validity, if known. Reference to where data collection forms can be found, if not in the protocol	11-14
	18b	Plans to promote participant retention and complete follow-up, including list of any outcome data to be collected for participants who discontinue or deviate from intervention protocols	11, 14
Data management	19	Plans for data entry, coding, security, and storage, including any related processes to promote data quality (eg, double data entry; range checks for data values). Reference to where details of data management procedures can be found, if not in the protocol	15

Statistical methods	20a	Statistical methods for analysing primary and secondary outcomes. Reference to where other details of the statistical analysis plan can be found, if not in the protocol	15
	20b	Methods for any additional analyses (eg, subgroup and adjusted analyses)	15
	20c	Definition of analysis population relating to protocol non-adherence (eg, as randomised analysis), and any statistical methods to handle missing data (eg, multiple imputation)	15
**Methods: Monitoring**			
Data monitoring	21a	Composition of data monitoring committee (DMC); summary of its role and reporting structure; statement of whether it is independent from the sponsor and competing interests; and reference to where further details about its charter can be found, if not in the protocol. Alternatively, an explanation of why a DMC is not needed	NA
	21b	Description of any interim analyses and stopping guidelines, including who will have access to these interim results and make the final decision to terminate the trial	NA
Harms	22	Plans for collecting, assessing, reporting, and managing solicited and spontaneously reported adverse events and other unintended effects of trial interventions or trial conduct	15
Auditing	23	Frequency and procedures for auditing trial conduct, if any, and whether the process will be independent from investigators and the sponsor	NA
**Ethics and dissemination**			
Research ethics approval	24	Plans for seeking research ethics committee/institutional review board (REC/IRB) approval	17
Protocol amendments	25	Plans for communicating important protocol modifications (eg, changes to eligibility criteria, outcomes, analyses) to relevant parties (eg, investigators, REC/IRBs, trial participants, trial registries, journals, regulators)	NA
Consent or assent	26a	Who will obtain informed consent or assent from potential trial participants or authorised surrogates, and how (see Item 32)	6
	26b	Additional consent provisions for collection and use of participant data and biological specimens in ancillary studies, if applicable	NA
Confidentiality	27	How personal information about potential and enrolled participants will be collected, shared, and maintained in order to protect confidentiality before, during, and after the trial	17

For peer review only - http://bmjopen.bmj.com/site/about/guidelines.xhtml

Declaration of interests	28	Financial and other competing interests for principal investigators for the overall trial and each study site	17
Access to data	29	Statement of who will have access to the final trial dataset, and disclosure of contractual agreements that limit such access for investigators	17
Ancillary and post-trial care	30	Provisions, if any, for ancillary and post-trial care, and for compensation to those who suffer harm from trial participation	NA
Dissemination policy	31a	Plans for investigators and sponsor to communicate trial results to participants, healthcare professionals, the public, and other relevant groups (eg, via publication, reporting in results databases, or other data sharing arrangements), including any publication restrictions	17
	31b	Authorship eligibility guidelines and any intended use of professional writers	NA
	31c	Plans, if any, for granting public access to the full protocol, participant-level dataset, and statistical code	NA
**Appendices**			
Informed consent materials	32	Model consent form and other related documentation given to participants and authorised surrogates	Appendix B, page 6
Biological specimens	33	Plans for collection, laboratory evaluation, and storage of biological specimens for genetic or molecular analysis in the current trial and for future use in ancillary studies, if applicable	NA

For peer review only

B. Participant information sheet and participant consent form

PARTICIPANT INFORMATION SHEET

"A tailored and targeted pharmacist intervention to improve adherence to antihypertensive drugs among patients with diabetes in Indonesia: a cluster randomized controlled trial"

This study is conducted by Sofa D. Alfian (PhD student at University of Groningen, the Netherlands), under daily supervision of Rizky Abdulah, PhD (Universitas Padjadjaran, Indonesia) and the supervision of Job F.M. van Boven, PhD (University Medical Center Groningen, Groningen, the Netherlands), Prof. Petra Denig (University Medical Center Groningen, Groningen, the Netherlands) and Prof. Eelko Hak (University of Groningen, Groningen, the Netherlands). You are invited to take part in a study that assesses different types of pharmacist services regarding the use of your antihypertensive drugs.

Objectives of the study

We want to compare the different types of pharmacist services on how you use your antihypertensive drugs, your blood pressure level, and your views about your antihypertensive drugs. We also want to know your opinion about the pharmacy service you received.

Participants

We are interested to include participants who:

- Are aged over 18 years
- Have type 2 diabetes
- Use antihypertensive drugs for at least three months

Procedure

The *Puskesmas* will be randomly assigned to different types of pharmacist services. The *Puskesmas* will have an equal chance of being placed in either group (like the flip of a coin). After you sign a written informed consent, you will receive guideline-based pharmacist counseling or patient-centred based counseling depending on your *Puskesmas*. Per *Puskesmas,* everyone will receive the same program. Your study participation will require three visits during the follow-up of 3 months with approximately up to 15-20 minutes per visit. We will

For peer review only - http://bmjopen.bmj.com/site/about/guidelines.xhtml

also ask you to complete a questionnaire. In addition, we will collect information from your medical record about your disease and other medication you are using.

Possible risks and inconvenience

There are no expected risks from participating in this study. However, up to 15-20 minutes of your time will be used during your regular visit to *Puskesmas*.

Benefits and compensation

There are no direct benefits of participation guaranteed to you in either group. Participation in either group will be at no cost to you. A modest compensation will be provided for your participation.

Participation and withdrawal from the study

Although it is important, your participation in this study is voluntary. You can change your mind at any time and you do not have to say why.

Confidentiality

All of the information you provide will be treated confidentially. A unique identifier will be used to match your data across your visits. Data will be stored securely and anonymously and only available to authorized individuals for analysis and reporting purpose only. Data will be published in a form that does not identify you in any way. Data will be stored for 15 years.

Whom to contact

If you have any concerns about this study that you think I can help you with, please feel free to contact me on: Sofa D. Alfian, phone: +62 85223 083 624, *email*: sofa.alfian@unpad.ac.id

If you would like to talk to someone who is not connected with the research, you may contact the Research Ethics Officer by *email:* etik.unpad@gmail.com

For peer review only - http://bmjopen.bmj.com/site/about/guidelines.xhtml

PARTICIPANT CONSENT FORM

I ________________________ (participant's name) agree to participate in the study "A tailored and targeted pharmacist intervention to improve adherence to antihypertensive drugs among patients with type 2 diabetes in Indonesia: a cluster randomized controlled trial" being conducted by Sofa D. Alfian of the University of Groningen for her PhD degree at the Groningen Research Institute of Pharmacy, Unit Pharmaco-Therapy, -Epidemiology & -Economics, The Netherlands.

By giving my consent, I acknowledge that:

- I have understood the Participant Information Sheet and have been given the opportunity to discuss the information and my involvement in the study.
- The procedures required for the study, the time involved (around 15-20 minutes per visit), and any inconvenience have been explained to me.
- I agree that information about my disease and other drugs I am using is collected from medical records, and is used in this study in a way that does not identify me.
- I understand that being in this study is voluntary.
- I understand that I am free to withdraw my participation from this study at any time I wish, without consequences, and without giving a reason.
- I agree that the research data gathered from this study may be published in a form that does not identify me in any way.
- I am aware that I can contact researcher (Sofa D. Alfian) if I have concerns about the research

Date ___/___/___

Signature,

For peer review only - http://bmjopen.bmj.com/site/about/guidelines.xhtml

C. Personalized leaflet

Name of patient : **Date:**
Agreed goal :
Date for next visit :

Your blood pressure lowering drugs			
Medicine name	When and how to take it?	What does it do?	Specific remarks

For peer review only

Reminders, habit-based strategies and/or involvement of family member

☐ Use pill boxes or a reminder app in your mobile phone.
☐ Make taking your blood pressure lowering drug(s) a part of your routine. Take it every day at around the same time. Choose a time that is easy to remember.

Coping plan (first visit) *	
IF it is (time of day): ……………………………………………………<br>And I am (where): ……………………………………………………<br>And I am (doing what): ……………………………………………………<br>THEN I will take (names of medication) ……………………………………………………<br>……………………………………………………	IF it is (time of day): ……………………………………………………<br>And I am (where): ……………………………………………………<br>And I am (doing what): ……………………………………………………<br>THEN I will take (names of medication) ……………………………………………………<br>……………………………………………………
**Follow-up after 1 month**	Succeeded ☐<br>If not, please explain why:<br>……………………………………………………

* Example: IF it is: first thing in the morning, and I am: in the kitchen, and I: am making my cup of tea, THEN: I will take my drug A

☐ Ask a family member to remind you to take your blood pressure lowering drug(s). I am going to ask: ……………………………………
☐ If you forgot to take the drug and it is less than 8 hours before your next dose, do not take a double dose of your blood pressure lowering drug(s) to make up for a forgotten dose. If it is more than 8 hours before your next dose, take the forgotten dose and take the next one as scheduled.

For peer review only - http://bmjopen.bmj.com/site/about/guidelines.xhtml

Knowledge about your blood pressure lowering drug(s)

- [ ] The purpose of your blood pressure lowering drug(s) is to reduce blood pressure level (short term effect) and to reduce the risk of developing heart disease in the future (long-term effect).
- [ ] Swallow your blood pressure lowering drug(s) with a drink of water.
- [ ] Common possible side effects of blood pressure lowering drug(s) are persistent dry cough, headache, dizziness, etc. If you get any side effects, please talk to your doctor or pharmacist. This includes any possible side effects not listed in this leaflet.

Motivation to take your blood pressure lowering drug(s)

- [ ] Your blood pressure lowering drug(s) only works when taken regularly as prescribed.
- [ ] Your blood pressure lowering drug(s) will not necessarily make you feel any different, but this does not mean it is not working.
- [ ] Do not stop taking your blood pressure lowering drug(s) without your doctor's consent unless you have an allergic reaction, e.g. rash or difficulty breathing. Abruptly stopping your blood pressure lowering drug(s) can cause a sudden increase in your blood pressure, which can be dangerous in some patients.
- [ ] Like all medicines, your blood pressure lowering drug(s) can cause side effects, but not everybody gets them. If you get any side effects, please talk to your doctor or pharmacist.
- [ ] Other:

____

____

____

Other drug-related problems

- [ ] If you have problem to refill your blood pressure lowering drug(s), please ask for help from your family or friend to accompany you to refill your medication.
- [ ] Other:

____

____

For peer review only

D. **Counseling protocol in the intervention group**

Baseline session

1. **Opening**

*I would like to start with reviewing some of your medications and then we can talk about your experiences of taking them. (*Complete the "Your blood pressure lowering drugs' list at the top of the Personalized Leaflet*)*

2. **Identify any possible barriers using the flowchart at baseline and provide the intervention based on the adherence intervention wheel**

Try to find out whether there are any barriers for not using the blood pressure lowering drug(s). Ask more if something is not clear. Once any barriers have been identified, deliver the intervention accordingly.

1. **Forgetfulness: setting coping plans and reminder tools**

Do not try to come directly with solutions and answers. Let the patient formulate his/her plans.

1. *How often do you forget to take your blood pressure lowering drug(s)?*
2. *Do you know when or why it happens that you forget to take your blood pressure lowering drug(s)?*

(Possible answers: being busy, not able to make taking the drug a part of their routine, difficult to remember when to take the drug, forgot to take the drug when traveling, or lack of family support. Patients may have other answers regarding the reason for forgetfulness, please provide and adapt the solution accordingly.)

3. *What time do you usually take each of your blood pressure lowering drug(s)?*
4. *What would you like to see different about your current situation?*

For peer review only - http://bmjopen.bmj.com/site/about/guidelines.xhtml

5. *I would start with a simple way for reminding you to take your medicines such as set an alarm on your mobile phone or set a coping plan.* (Give the personalized leaflet and ask patients to write down their plans in the box Reminders).
6. *Could you ask your family to remind you to take your blood pressure lowering drug(s) regularly (if so, ask patient to write down name of family member in the box Involvement of family member)?*
7. Explain what to do when a dose is forgotten, for example*: If you forget to take a dose of your blood pressure lowering drug, take it as soon as you remember. However, if it less than 8 hours before your next dose is scheduled, skip the missed dose(s) and go back to your regular dosing schedule. Do not take a double dose of your blood pressure lowering drug to make up for a forgotten dose.*

2. Lack of knowledge: counseling and education

1. *Do you understand why you have to take your blood pressure lowering drug(s)?*
   a. If yes: *Can you explain this to me?*
   b. If no or insufficient: explain the purpose of blood pressure lowering drug(s) regarding short (blood pressure) and long-term effects (prevention of heart attack and stroke); when relevant also explain which side effects can occur and how to deal with them when they occur. For example:

For peer review only

i. Short term effect: *The purpose of your blood pressure lowering drug(s) is to reduce your blood pressure by relaxing or widening your blood vessels, making your heartbeat more slowly, or flushing excess water and salt from the body through your pee.*

ii. Long term effect*: The purpose of your blood pressure lowering drug(s) is to reduce the risk of developing heart disease in the future, such as heart attack and stroke.*

2. *Do you understand how you have to take your blood pressure lowering drug(s)?*
   a. If yes: *Can you explain this to me?*
   b. If no or insufficient: Explain when to take and how much to take.
3. *Could you explain what we have talked about so far?*
4. *Do you have more questions?*

3. Lack of motivation: counseling

1. *Do your blood pressure lowering drug(s) bother you in any way?*

   If yes: Explore in what way the drug bother the patient. If it is related to the high concerns and low necessity, continue with the following questions.

2. *Do you think that it is important to use blood pressure lowering drug(s)?*
   a. If yes: *Can you explain this to me?*
   b. If no or insufficient: Explain that the blood pressure lowering drug(s) only works when taken as prescribed. If patients answer that they did not feel anything changed after taking the drug, explain that blood pressure

For peer review only - http://bmjopen.bmj.com/site/about/guidelines.xhtml

lowering drug(s) will not necessarily make them feel any different, but this does not mean the drug is not working. When relevant, also explain the importance of the blood pressure lowering drug(s) in reducing blood pressure level and prevent future cardiovascular diseases.

3. *Do you have any concerns when using blood pressure lowering drug(s)?*
   a. If no: Continue with the following question.
   b. If yes: Explore what the concerns are. If it is related to side effects, try to reduce it by explaining the common possible side effects which is not dangerous and by convincing patients that not everybody experienced the side effects. For example, start with this statement:
      Most people will be able to take their medicines without any problems. However, a few people can have side effects from their medicines. Everyone is different, and there is no way to tell whether you will have a side effect from your medicine. Most people who have side effects will feel them soon after they start to take a new medicine, or a higher dose of their medicine. (Of note, most of the more serious side effects of blood pressure lowering drug(s) are rare, for example, severe allergic reactions may affect up to 1 in 1,000 people or severe skin disorders -a sudden, unexpected rash or burning- may affect up to 1 in 10,000 people).

      Continue with this statement to reduce concerns about becoming too dependent on blood pressure lowering drug(s):

For peer review only - http://bmjopen.bmj.com/site/about/guidelines.xhtml

High blood pressure is a long-term problem that requires long-term treatment to keep it under control. For this reason, most people need to take tablets to keep their blood pressure down for the rest of their lives. However, a few people are able to bring their blood pressure down by really sticking to changes in their lifestyle, take the drug as prescribed, and later may be able to reduce the dose or some of their tablets.

4. *Are you worried about any side effects in the long term?*
   a. If no: Continue with the following question.
   b. If yes: Explain that some side effects may disappear over time, or patients may get used to them. Most side effects of blood pressure lowering drug(s) are not dangerous. Patients can always talk to doctor or pharmacist. For example:

      If you start to feel different after taking your blood pressure lowering drug(s) in the long-term, please talk to your doctor or to me. Your doctor may be able to change your dose, or try a different medicine.

4. Other problems: explore and offer possible solutions

1. *Do you have any side effects that you think may be caused by your blood pressure lowering drug(s)?*

   If yes: please call the Puskesmas or come over to Puskesmas.
2. *Do you have any other problem with taking or collecting your blood pressure lowering drug(s)?*
   a. If yes: Possible answers are due to experiencing side effect, polypharmacy, financial issue, lack of access to CHC, or the use of traditional medicine.

For peer review only - http://bmjopen.bmj.com/site/about/guidelines.xhtml

Patients may have other answers that are not listed here. Try to offer solutions according to their answer, for example, if patients have a difficulty to refill the drug in time, suggest them to ask for help from family of friend to accompany them to refill the blood pressure lowering drug(s).

b. If no: *Can you explain why you sometimes/often/always do not take your blood pressure lowering drug(s)?* (Some patients take less medication or lower dosages as 'self-management'; such patients may have good knowledge and motivation*)*

3. Closing

At the end of each counseling, involve patients in a goal setting process, for example to reduce forgetfulness. Ask their current blood pressure level and take it into account when discussing the goal. If patients already have a controlled blood pressure, explain that they can receive a reduced doses or reduced number of blood pressure lowering drug(s) (when relevant) in the future if they can maintain the blood pressure level under control. This must be done after discussing it first with the responsible doctor. After that, write the agreed goal at the top of the personalized leaflet for patients to take home. Please remind patients to take his/her leaflet in the next visit, and **make a copy of the leaflet for your own and the researchers to use**.

Follow-up session

1. Review and discuss patient's implementation and experiences with the discussed plans and recommendations.

For peer review only - http://bmjopen.bmj.com/site/about/guidelines.xhtml

1. For those who already adherent in the follow-up session: *You should be proud of all effort you have put into taking your blood pressure lowering drug(s) as prescribed to you.*
2. *What is your experience so far? How is it going?*

2. Identify all further barriers that were not yet addressed during the first session using the <u>flowchart at 1-month follow up visit</u> and provide the intervention based on the <u>adherence intervention wheel.</u>

Try to find out whether there are still any barriers for not using the blood pressure lowering drug(s). Ask more if something is not clear. Once any barriers have been identified, deliver the intervention accordingly (see baseline session).

For peer review only

A. SPIRIT checklist

SPIRIT
STANDARD PROTOCOL ITEMS: RECOMMENDATIONS FOR INTERVENTIONAL TRIALS

For peer review only

Section/item	Item No	Description	Reported on page
**Administrative information**			
Title	1	Descriptive title identifying the study design, population, interventions, and, if applicable, trial acronym	1
Trial registration	2a	Trial identifier and registry name. If not yet registered, name of intended registry	2
	2b	All items from the World Health Organization Trial Registration Data Set	4-20
Protocol version	3	Date and version identifier	NA
Funding	4	Sources and types of financial, material, and other support	17
Roles and responsibilities	5a	Names, affiliations, and roles of protocol contributors	1, 17
	5b	Name and contact information for the trial sponsor	NA
	5c	Role of study sponsor and funders, if any, in study design; collection, management, analysis, and interpretation of data; writing of the report; and the decision to submit the report for publication, including whether they will have ultimate authority over any of these activities	17
	5d	Composition, roles, and responsibilities of the coordinating centre, steering committee, endpoint adjudication committee, data management team, and other individuals or groups overseeing the trial, if applicable (see Item 21a for data monitoring committee)	NA
**Introduction**			

For peer review only - http://bmjopen.bmj.com/site/about/guidelines.xhtml

Background and rationale	6a	Description of research question and justification for undertaking the trial, including summary of relevant studies (published and unpublished) examining benefits and harms for each intervention	4-5
	6b	Explanation for choice of comparators	10
Objectives	7	Specific objectives or hypotheses	5
Trial design	8	Description of trial design including type of trial (eg, parallel group, crossover, factorial, single group), allocation ratio, and framework (eg, superiority, equivalence, non-inferiority, exploratory)	6,7
**Methods: Participants, interventions, and outcomes**			
Study setting	9	Description of study settings (eg, community clinic, academic hospital) and list of countries where data will be collected. Reference to where list of study sites can be obtained	6
Eligibility criteria	10	Inclusion and exclusion criteria for participants. If applicable, eligibility criteria for study centres and individuals who will perform the interventions (eg, surgeons, psychotherapists)	6,7
Interventions	11a	Interventions for each group with sufficient detail to allow replication, including how and when they will be administered	7-10
	11b	Criteria for discontinuing or modifying allocated interventions for a given trial participant (eg, drug dose change in response to harms, participant request, or improving/worsening disease)	NA
	11c	Strategies to improve adherence to intervention protocols, and any procedures for monitoring adherence (eg, drug tablet return, laboratory tests)	14
	11d	Relevant concomitant care and interventions that are permitted or prohibited during the trial	7
Outcomes	12	Primary, secondary, and other outcomes, including the specific measurement variable (eg, systolic blood pressure), analysis metric (eg, change from baseline, final value, time to event), method of aggregation (eg, median, proportion), and time point for each outcome. Explanation of the clinical relevance of chosen efficacy and harm outcomes is strongly recommended	11-13
Participant timeline	13	Time schedule of enrolment, interventions (including any run-ins and washouts), assessments, and visits for participants. A schematic diagram is highly recommended (see Figure)	Figure 1
Sample size	14	Estimated number of participants needed to achieve study objectives and how it was determined, including clinical and statistical assumptions supporting any sample size calculations	14

Recruitment	15	Strategies for achieving adequate participant enrolment to reach target sample size	14
**Methods: Assignment of interventions (for controlled trials)**			
Allocation:			
Sequence generation	16a	Method of generating the allocation sequence (eg, computer-generated random numbers), and list of any factors for stratification. To reduce predictability of a random sequence, details of any planned restriction (eg, blocking) should be provided in a separate document that is unavailable to those who enrol participants or assign interventions	7
Allocation concealment mechanism	16b	Mechanism of implementing the allocation sequence (eg, central telephone; sequentially numbered, opaque, sealed envelopes), describing any steps to conceal the sequence until interventions are assigned	7
Implementation	16c	Who will generate the allocation sequence, who will enrol participants, and who will assign participants to interventions	7
Blinding (masking)	17a	Who will be blinded after assignment to interventions (eg, trial participants, care providers, outcome assessors, data analysts), and how	7
	17b	If blinded, circumstances under which unblinding is permissible, and procedure for revealing a participant's allocated intervention during the trial	NA
**Methods: Data collection, management, and analysis**			
Data collection methods	18a	Plans for assessment and collection of outcome, baseline, and other trial data, including any related processes to promote data quality (eg, duplicate measurements, training of assessors) and a description of study instruments (eg, questionnaires, laboratory tests) along with their reliability and validity, if known. Reference to where data collection forms can be found, if not in the protocol	11-14
	18b	Plans to promote participant retention and complete follow-up, including list of any outcome data to be collected for participants who discontinue or deviate from intervention protocols	11, 14
Data management	19	Plans for data entry, coding, security, and storage, including any related processes to promote data quality (eg, double data entry; range checks for data values). Reference to where details of data management procedures can be found, if not in the protocol	15
Statistical methods	20a	Statistical methods for analysing primary and secondary outcomes. Reference to where other details of the statistical analysis plan can be found, if not in the protocol	15

	20b	Methods for any additional analyses (eg, subgroup and adjusted analyses)	15
	20c	Definition of analysis population relating to protocol non-adherence (eg, as randomised analysis), and any statistical methods to handle missing data (eg, multiple imputation)	15
**Methods: Monitoring**			
Data monitoring	21a	Composition of data monitoring committee (DMC); summary of its role and reporting structure; statement of whether it is independent from the sponsor and competing interests; and reference to where further details about its charter can be found, if not in the protocol. Alternatively, an explanation of why a DMC is not needed	NA
	21b	Description of any interim analyses and stopping guidelines, including who will have access to these interim results and make the final decision to terminate the trial	NA
Harms	22	Plans for collecting, assessing, reporting, and managing solicited and spontaneously reported adverse events and other unintended effects of trial interventions or trial conduct	15
Auditing	23	Frequency and procedures for auditing trial conduct, if any, and whether the process will be independent from investigators and the sponsor	NA
**Ethics and dissemination**			
Research ethics approval	24	Plans for seeking research ethics committee/institutional review board (REC/IRB) approval	17
Protocol amendments	25	Plans for communicating important protocol modifications (eg, changes to eligibility criteria, outcomes, analyses) to relevant parties (eg, investigators, REC/IRBs, trial participants, trial registries, journals, regulators)	NA
Consent or assent	26a	Who will obtain informed consent or assent from potential trial participants or authorised surrogates, and how (see Item 32)	6
	26b	Additional consent provisions for collection and use of participant data and biological specimens in ancillary studies, if applicable	NA
Confidentiality	27	How personal information about potential and enrolled participants will be collected, shared, and maintained in order to protect confidentiality before, during, and after the trial	17
Declaration of interests	28	Financial and other competing interests for principal investigators for the overall trial and each study site	17

For peer review only

Access to data	29	Statement of who will have access to the final trial dataset, and disclosure of contractual agreements that limit such access for investigators	17
Ancillary and post-trial care	30	Provisions, if any, for ancillary and post-trial care, and for compensation to those who suffer harm from trial participation	NA
Dissemination policy	31a	Plans for investigators and sponsor to communicate trial results to participants, healthcare professionals, the public, and other relevant groups (eg, via publication, reporting in results databases, or other data sharing arrangements), including any publication restrictions	17
	31b	Authorship eligibility guidelines and any intended use of professional writers	NA
	31c	Plans, if any, for granting public access to the full protocol, participant-level dataset, and statistical code	NA
**Appendices**			
Informed consent materials	32	Model consent form and other related documentation given to participants and authorised surrogates	Appendix B, page 6
Biological specimens	33	Plans for collection, laboratory evaluation, and storage of biological specimens for genetic or molecular analysis in the current trial and for future use in ancillary studies, if applicable	NA

For peer review only
